# Permeation thresholds for hydrophilic small biomolecules across microvascular and epithelial barriers are predictable on basis of conserved biophysical properties

**DOI:** 10.1186/s40203-015-0009-y

**Published:** 2015-05-03

**Authors:** Hemant Sarin

**Affiliations:** Freelance Investigator in Translational Science and Medicine, Charleston, WV USA

**Keywords:** Octanol-to-water partition coefficient, Molecular size, Cationic biomolecule, Anionic biomolecule, Cationoneutral biomolecule, Polyneutral biomolecule, Molecular charge, Cations, Anions, Molecular toxicology

## Abstract

**Purpose:**

Neutral small hydrophiles are permeable to varying degrees, across the aqueous pores of phospholipid bilayer protein channels, with their potential for permeation into cells being predictable, on the basis of hydrophilicity and size. Here, it is hypothesized that permeation thresholds for small hydrophiles, across capillary zona occludens tight junction and inter-epithelial junction pore complexes are predictable, on the basis of predicted hydrophilicity in context of predicted molecular size and charge distribution, as are those of cations and anions, on the basis of predicted ionization in context of predicted atomic size.

**Methods:**

Small hydrophiles are categorized by charge distribution. 2-dimensional plots of predicted hydrophilic octanol-to-water partition coefficient (HOWPC; unitless) and predicted van der Waals diameter (vdWD; nm) are generated for each category. The predicted HOWPC-to-vdWD ratio (*nm*^*-1*^), and vdWDs for permeable hydrophile at the maximum and minimum HOWPC-to-vdWD, vdWD @ MAXimum HOWPC-to-vdWD and vdWD @ MINimum HOWPC-to-vdWD are determined. For cations and anions, the ionization-to-atomic diameter ratios (CI or AI-to-AD ratios; nm^-1^) are determined.

**Results:**

Per sizes of mixed and pure polyneutral hydrophiles, the permeation size maximum for hydrophiles across tight junction pore complexes is >0.69 ≤ 0.73 nanometers and across inter-epithelial junction pore complexes is ≥ 0.81 nanometers. For hydrophiles with anionicity or cationicity, the vdWDs @ MAXimum HOWPC-to-vdWD are less than those of mixed and polyneutral hydrophiles across both tight and inter-epithelial junctions, ranges specific to category and junction type. For cations, the permeation threshold across tight junctions is between the CI-to-AD ratio of Na+ (+2.69 nm^-1^) and CH3-Hg+ (+2.36 nm^-1^), with CH3-Hg+ and K+ (+2.20 nm^-1^) being permeable; and for divalent cations, the threshold across inter-epithelial junctions is between the CI-to-AD ratio of Mg2+ (+6.25 nm^-1^) and Ca2+ (+5.08 nm^-1^) , Ca2+ being semi-permeable. For anions, the permeation threshold across tight junctions is between the AI-to-AD ratio of Cl- (-4.91 nm^-1^) and Br- (-4.17 nm^-1^), and the threshold across inter-epithelial junctions is between the AI-to-AD ratio of F- (-7.81 nm^-1^) and Cl- (-4.91 nm^-1^).

**Conclusions:**

*In silico* modeling reveals that permeation thresholds, of small molecule hydrophiles, cations and anions across junctional pore complexes, are conserved in the physiologic state.

**Electronic supplementary material:**

The online version of this article (doi:10.1186/s40203-015-0009-y) contains supplementary material, which is available to authorized users.

## Background

Over the years the transcapillary permeation potential of hydrophilic macromolecules (>2 nanometers), small biomolecules and electrolytes has been studied utilizing various *in situ*, *in vivo* and *ex vivo* approaches (Pappenheimer 1953; Grotte 1955, 1956; Renkin 1977; Crone 1963; Palade 1961; Karnovsky 1968; Casley-Smith 1967; Bearer et al. 1985; Michel 1979, 1996). In more recent years, blood capillaries have been classified with attention to differences in the aqueous pore populations of their endothelial cell lining with respect to upper limits of pore size to the transcapillary permeation of native macromolecules with neutral exteriors (Sarin 2010), which is the important determinant of the specificity of biomolecular action in the physiologic state for tissue differentiation and maintenance.

Two main aqueous pore populations exist, which are the trans-endothelial pores through individual endothelial cells that are either openly fenestrated or fenestrated with intervening diaphragms of endothelial cell membrane, and the inter-endothelial cell pores of the series of inter-endothelial junctional complexes in-between juxtaposed endothelial cells that consist of either macula occludens loose junction pore complexes or zona occludens tight junction pore complexes (Sarin 2010). For tissue-organs with secretory functions, in the case of the liver, openly fenestrated trans-endothelial pores exist in the sinusoidal blood capillaries across which secretion of large globular proteins occurs, in the case of the myeloid bone marrow, transient openly fenestrated trans-endothelial pores exist in the sinusoidal blood capillaries across which secretion of hematopoietic cells and small growth factors occurs, and in the case of the endocrine glands, diaphragm fenestrated trans-endothelial pores exist in the sinusoidal-type blood capillaries across which the secretion of subunits of globular protein occurs, with upper limits of pore size of these sinusoidal blood capillary and sinusoid type blood capillaries approximately 100 nanometers, 7 nanometers and 6 nanometers to the passage of native macromolecules with neutral exteriors (Sarin 2010). In the case of these secretory tissues, the liver, myeloid bone marrow and endocrine glands, these have dual blood capillary circulations; the primary blood capillary beds of these tissue-organs is a pre-capillary arteriole sphincter-regulated circulation of continuous blood capillaries with inter-endothelial pores of a series of zona occludens tight junction pore complexes, which are uniformly restrictive to the passage of macromolecules; in contrast, the blood capillary network of cardiac and skeletal muscle tissue-organs is a circulation of continuous blood capillaries with inter-endothelial pores of a series of macula occludens loose junction pore complexes, which have an upper limit of pore size of approximately 4 nanometers to the passage of native macromolecules with neutral exteriors (Sarin 2010). Examples of tissue-organs that have singular blood capillary circulations include the brain and the spinal cord of central nervous system and the endoneurium of peripheral nerves, which are supplied by continuous blood capillaries with inter-endothelial pores of the tight junction pore complex sub-type that have an estimated upper limit of pore size in the range of 0.5 to 1 nanometer to hydrophilic small molecules (Sarin 2010), but for which the upper limit of pore size has not yet been determined with respect to differences in the biophysical properties of hydrophilic small biomolecules, cations and anions.

In a tissue-organ-based biological system in the physiologic state, in order for the uptake hydrophilic small molecules, cations and anions into system blood circulation, the entry of hydrophilic small molecules, cations and anions into the system must occur through epithelial barriers (Farquhar and Palade 1963; Madara and Pappenheimer 1987; Pappenheimer 1988; Brightman and Reese 1969), for then, the entry into the blood capillary beds, which is in series: This makes it important to study the thresholds of both sets of barriers, epithelial and endothelial, to hydrophilic small molecule, cation and anion permeation. To-date, however, the trans-epithelial and transcapillary permeability thresholds of relatively a few hydrophilic small biomolecules have been determined, which has been via measurements of diffusional permeability reflection coefficients either *in situ* or *in vivo* (Crone 1963; Fenstermacher and Johnson 1966; Davson 1955; Davson and Welch 1971; Seiguer and Mancini 1971; Sorensen 1974; Michel and Curry 1999), without emphasis on how the amount or character of hydrophilicity of a small biomolecule influences its potential for its permeation across blood capillary walls in context of the potential of toxicity to the biological system.

Most recently, the conserved biophysical determinants for the interactions of small hydrophilic biomolecules, cations and anions in the biological system in the physiologic state have been elucidated , which, for biomolecules categorized on the basis of character of charge distribution over molecular space, are the relative hydrophilicity of a biomolecule, as per the predicted hydrophilic octanol-to-water partition coefficient (HOWPC; unitless), in context of molecular size, as per the predicted van der Waals diameter (vdWD; nm), considered in terms of the predicted hydrophilic octanol-to-water partition coefficient-to-molecular diameter ratio (HOWPC-to-vdWD ratio; *nm*^*-1*^). Furthermore, in the case of cations and anions, it has been observed that interactions with cell membrane protein aqueous channels can also be predicted on the basis of relative cationicity as per its predicted Cationization-to-Atomic Diameter ratio (CI-to-AD ratio; nm^-1^) and the predicted Anionization-to-Atomic Diameter ratio (AI-to-AD ratio; nm^-1^) (Sarin H. Biological Function is Conserved in the Physiologic State [Submitted]).

By understanding biomolecular permeability on the basis of the conserved biophysical properties of small molecule hydrophiles, it would be possible to determine accurately the permeation potential of a cation or anion as well as that of a small hydrophilic biomolecule, stratified as, anionic, anionic-cataniononeutral, pure polyneutral, neutral-cataniononeutral/cataniononeutral, mixed polyneutral, neutral, cationic, cationic-cataniononeutral and cationic-anionic, across endothelial and epithelial barriers. Based on such determinations, it will be possible to predict *a priori* exactly the nature of biodistribution and biocompartmentalization, in context of the potential for toxicity, including at what dose it is likely to occur. Therefore, in this research study, the permeation potentials of hydrophilic small molecules, cations and anions across junctional pore complexes of microvascular capillary and epithelial barriers are determined *in silico* by considering character of hydrophilicity and its distribution over molecular space, while taking into account the presence of cationicity, anionicity, (poly)neutrality and the combination thereof. The mechanisms underlying toxicity to microvascular blood capillary and epithelial barriers and tissue spaces are also explored.

## Methods

### Data acquisition and determination of principal components for analysis for biomolecules, cations and anions

Hydrophilic small biomolecules endogenous to the biological system including carboxylic acids, sugars, nitrogenous bases, metabolites and breakdown products, amino acids, neurotransmitters, monovalent cations, divalent cations, trivalent cations and heavy metals, and anions were identified for the database. Some non-endogenous hydrophilic small molecule therapeutics with known permeability coefficients were included to complete the database, which also serve to illustrate the utility of the methodology.

Freely available and validated online biochemical molecule databases including http://Chemicalize.org and http://ChemSpider.com were utilized for determinations of molecular structure and ionization state at physiologic pH of ~7.4. Biomolecular configurations were assessed for polar surface area, while paying close attention to the type of covalent bonds within the structure, including the presence or absence of associated molecular charge, which were: Hydrogen [0], Halogen [0], Hydroxyl [0,-1], Phosphate [-1,-2], Carboxyl [-1], Carbonyl [C=O] &/or Amine [0, +1]: primary, secondary, tertiary or quaternary [+1]), & in the case of the presence of functional bond(s), whether amide or ester.

The online databases were also utilized for determinations of: (1) the predicted hydrophilic octanol-to-water partition coefficient (poHOWPC or HOWPC; unitless), whereby the predicted Log Pow is the applied value for biomolecules in the un-ionized state, and the Log Dow is the predicted value for biomolecules in the ionized state, which are both based on the predicted hydrophilic octanol-to-water partition coefficient itself, and utilized for the calculation of the predicted hydrophilic octanol-to-water partition coefficient-to-molecular diameter ratio (HOWPC-to-vdWD ratio; *nm*^*-1*^); (2) the predicted van der Waals Diameter (vdWD; nm) as the measure of estimated molecular size, which is based on the predicted spherical van der Waals volume; and (3) the atomic radii (nm). For cations and anions, the predicted cationization-to-atomic diameter ratio (CI-to-AD ratio; nm^-1^) and anionization-to-atomic Diameter ratio (AI-to-AD ratio; nm^-1^) were calculated.

To characterize hydrophilic small molecule permeation thresholds per category (anionic, anionic-cataniononeutral, pure polyneutral, neutral-cataniononeutral/cataniononeutral, mixed polyneutral, neutral, cationic, cationic-cataniononeutral and cationic-anionic) the following parameters were determined for each hydrophile category: (1) hydrophilic octanol-to-water partition coefficient-to-molecular diameter ratio (HOWPC-to-vdWD ratio; *nm*^*-1*^); (2) van der Waals Diameter (vdWD; nm) for a permeable hydrophile at the maximum hydrophilic octanol-to-water partition coefficient-to-molecular diameter ratio (vdWD @ MAXimum HOWPC-to-vdWD; nm), which represents the molecular size at which a hydrophile is permeable at maximum hydrophilicity; and (3) van der Waals Diameter (vdWD) for a permeable hydrophile at the minimum hydrophilic octanol-to-water partition coefficient-to-molecular diameter ratio (vdWD @ MINimum HOWPC-to-vdWD; nm), the maximum molecular size at which a hydrophile is permeable.

### Two-dimensional (2-D) principal component plots of biomolecule Octanol-to-water partition coefficients versus van der Waals diameters

2-D principal component plots of biomolecule predicted overall hydrophilicity octanol-to-water partition coefficient (poHOWPC; HOWPC; unitless) represented on the y-axis versus their van der Waals Diameters (vdWDs; nm) represented on the x-axis were generated for each category to visually separate out the relative contribution of a biomolecule’s overall hydrophilicity to that of its molecular size in each category with respect to tight junction and inter-epithelial junction pore complexes. For each category, these plots were analyzed via visual parabolic extrapolation with respect to knowledge of the published permeabilities of a certain subset of the biomolecules to tight junctions and inter-epithelial junctions, as referenced in the literature cited.

### Classification of molecular charge

The presence of charge and its distribution over biomolecular space was determined based on visual inspection of 2-D molecular structures. A classification scheme was developed for the characterization of molecular charge over molecular space. The classification scheme that is applied for delineation of charge distribution within a small biomolecule is as follows:

I. No overall charge: Neutral (0), PolyNeutral (Poly –n);
II. Sufficient molecular space separation of charge (S) is defined as the presence of non-focal charges separated over a non-flexible rigid covalent frame in molecular space, either in the form of sufficiently separated attractive + and – charges that result in sufficiently separated contributions of cationicity (+) and of anionicity (-), which result in a molecular dipole moment, or in insufficiently separated equivalent positive charges (+ +) or negative charges (- -) that result in an insufficiently separated synergistic contributions of cationicity (+) or anionicity (-) in electrical polarity.
III. Insufficient molecular space separation (IS) is defined as the presence of focal charge in molecular space, either in the form of insufficiently separated attractive + and – charge that results in insufficiently separated contributions of cationicity (+) and of anionicity (-), which results in electroneutral polarity and dilution of overall charge effect, or insufficiently separated equivalent positive charges (+ +) or negative charges (- -) that results in insufficiently separated synergistic contributions of cationicity (+) or anionicity (-) and electrical polarity.
  (A) Anionic-Neutral (1-, 0), Anionic-Anionic (S 1-, 1-) and PolyAnionic (Poly –n in molecule space)
  (B) Cationic-Neutral (1+, 0); Cationic-Cationic (S 1+, 1+)
  (C) Cationic-Anionic (PS 1+, 1-), and if non-rigid (NR) intervening bonds, then Cationic-Anionic NR (NR PS 1+, 1-), which results in partial sufficient (PS) separation of attractive charges
  (D) Cataniononeutral: Neutral-Cataniononeutral (0 IS 1+ 1-) and if no neutral group, then IS 1+, 1-; Anionic-Cataniononeutral (S 1- IS 1+ 1-) and Cationic-Cataniononeutral (S 1+ IS 1+, 1-)

## Results

### Hydrophilicity octanol-to-water partition coefficient in context of molecular size and the ratio

The predicted overall hydrophilicity octanol-to-water partition coefficient (poHOWPC or HOWPC), the predicted coefficient for the whole hydrophile, represents the presence of molecular surface area hydrophilicity due to the presence of functional groups of hydrophilic character, both those that are non-charged, hydroxyl (OH), sulphur (SH), carbonyl (C=O) and amide (N=O), and charged, O-, S-, COO- and N+, is the measure of overall hydrophilicity (Additional files 1, 2, 3, 4, 5, 6, 7, 8, 9, 10, 11, 12 and 13, Panels A and B). The van der Waals Diameter (vdWD; nm) represents molecular size, and consistently represents the hydrodynamic diameters of a subset of hydrophiles of known hydrodynamic diameters (Additional files 1, 2, 3, 4, 5, 6, 7, 8, 9: Panels A and B; Additional files 12, 13 and 14), with only small deviation from the line of unity upon regression analysis with similar slope but lower intercept for referenced biomolecules’ hydrodynamic diameters (unpublished result). The predicted hydrophilicity octanol-to-water partition coefficient (HOWPC)-to-van der Waals Diameter (vdWD) ratio (HOWPC—to-vdWD ratio; *nm*^*-1*^) represents the overall hydrophilicity of small molecule hydrophiles, irrespective of category (Additional files 1, 2, 3, 4, 5, 6, 7, 8, 9: Panels A and B; Additional files 12, 13 and 14).

**Figure 1:**
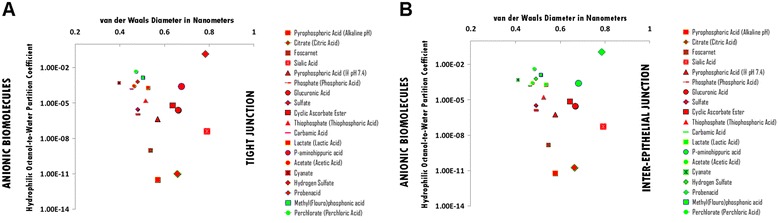
Panel **A**, Anionic Biomolecule Permeation across Tight Junction Pore Complexes; Panel **B**, Anionic Biomolecule Permeation across Inter-Epithelial Pore Complexes. Hydrophilic Octanol-to-Water Partition Coefficient (Y-axis) and van der Waals Diameter in Nanometers; Green = Permeable; Red – Not Permeable.

**Figure 2:**
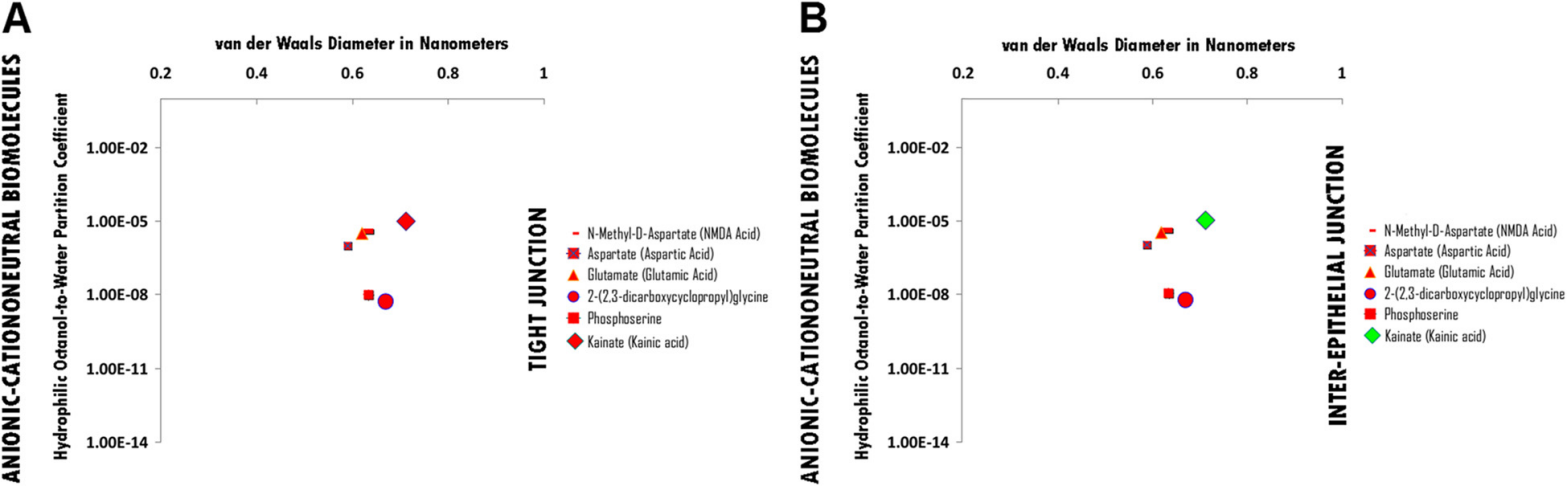
Panel **A**, Anionic-Cationoneutral Biomolecule Permeation across Tight Junction Pore Complexes ; Panel **B**, Anionic-Cationoneutral Biomolecule Permeation across Inter-Epithelial Pore Complexes. Hydrophilic Octanol-to-Water Partition Coefficient (Y-axis) and van der Waals Diameter in Nanometers; Green = Permeable; Red – Not Permeable.

**Figure 3:**
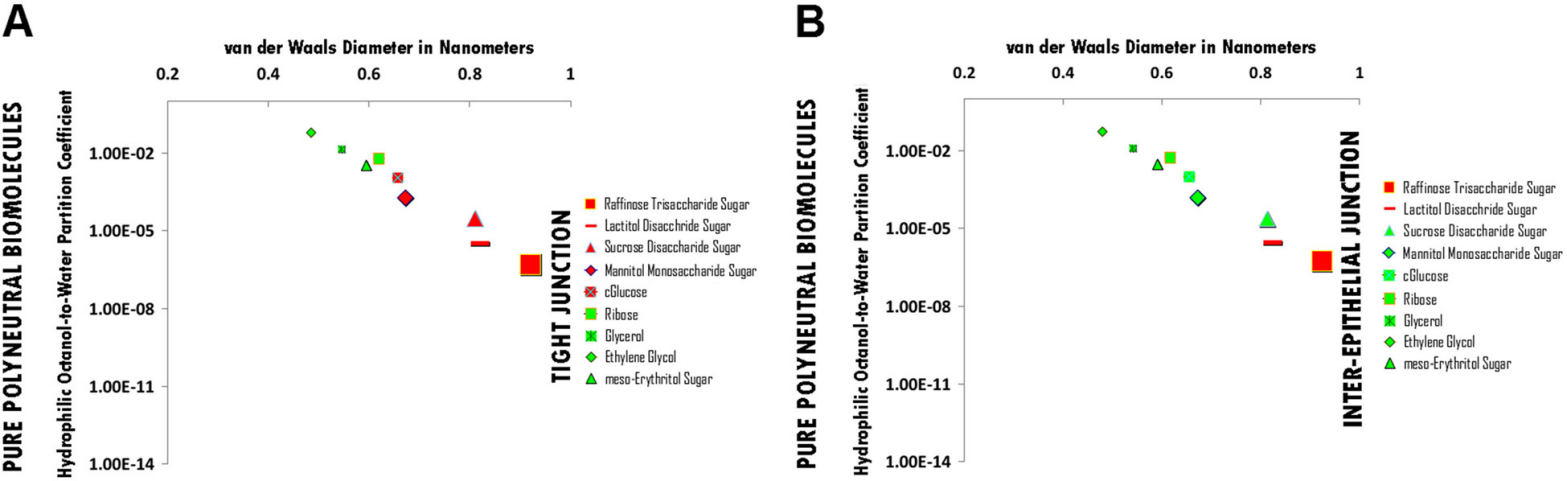
Panel **A**, Pure Polyneutral Biomolecule Permeation across Tight Junction Pore Complexes ; Panel **B**, Pure Polyneutral Biomolecule Permeation across Inter-Epithelial Pore Complexes. Hydrophilic Octanol-to-Water Partition Coefficient (Y-axis) and van der Waals Diameter in Nanometers; Green = Permeable; Red – Not Permeable.

**Figure 4:**
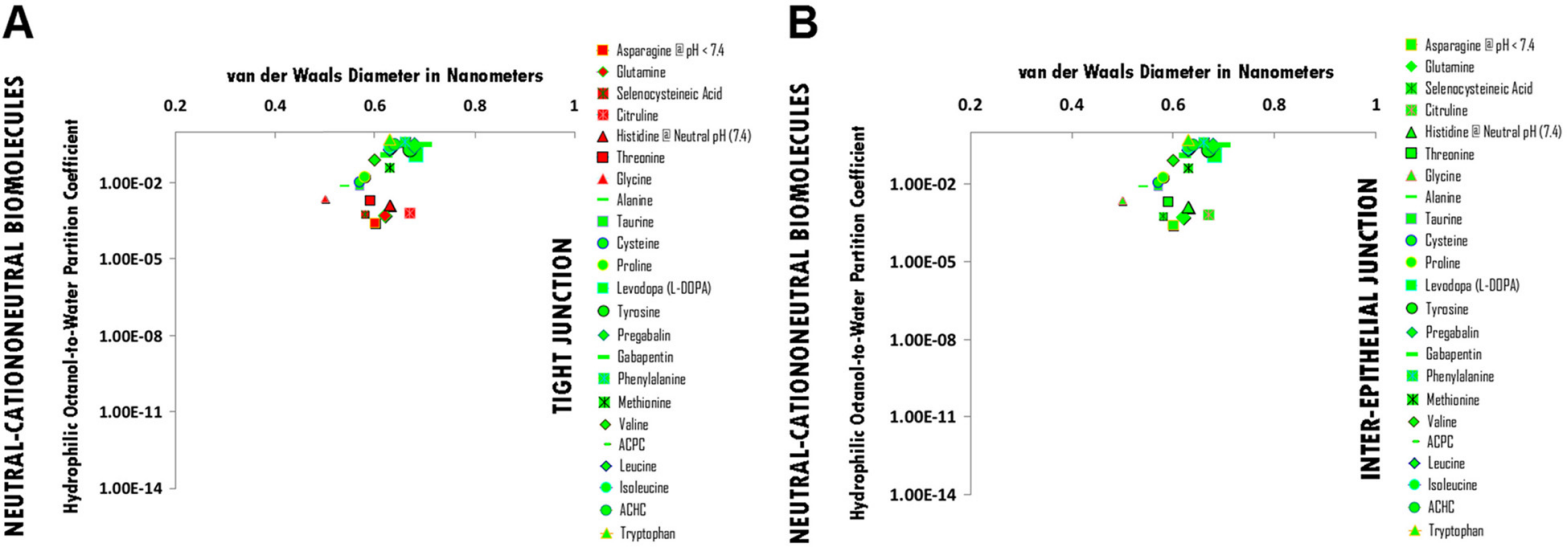
Panel **A**, Neutral-Cationoneutral Biomolecule Permeation across Tight Junction Pore Complexes ; Panel **B**, Neutral-Cationoneutral Biomolecule Permeation across Inter-Epithelial Pore Complexes. Hydrophilic Octanol-to-Water Partition Coefficient (Y-axis) and van der Waals Diameter in Nanometers; Green = Permeable; Red – Not Permeable.

**Figure 5:**
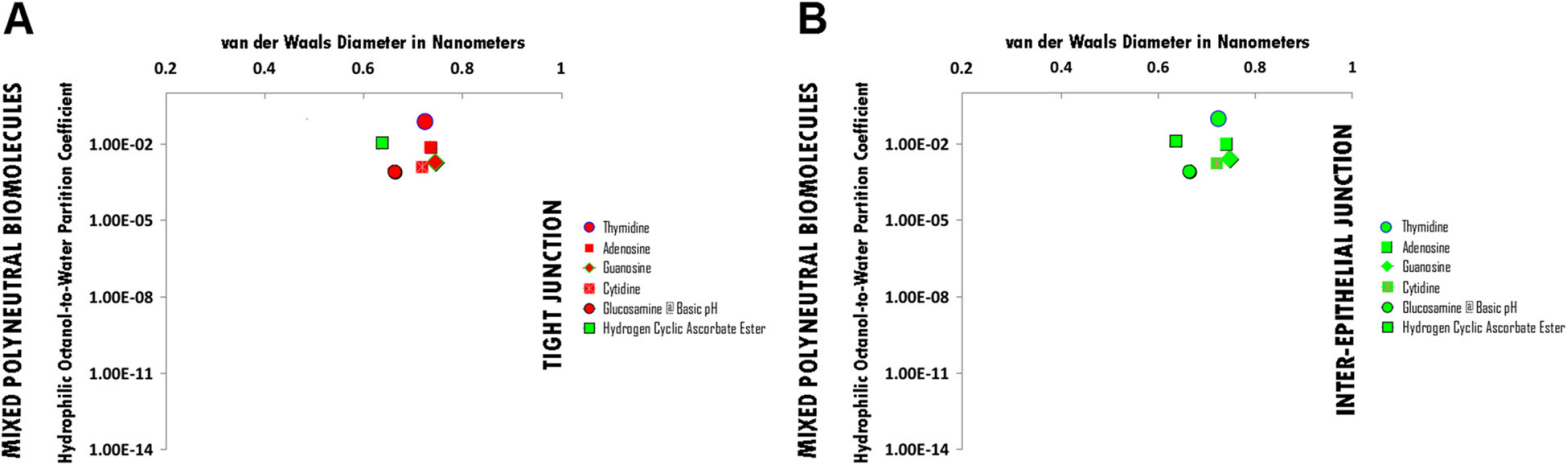
Panel **A**, Mixed Polyneutral Biomolecule Permeation across Tight Junction Pore Complexes; Panel **B**, Mixed Polyneutral Biomolecule Permeation across Inter-Epithelial Pore Complexes. Hydrophilic Octanol-to-Water Partition Coefficient (Y-axis) and van der Waals Diameter in Nanometers; Green = Permeable; Red – Not Permeable.

**Figure 6:**
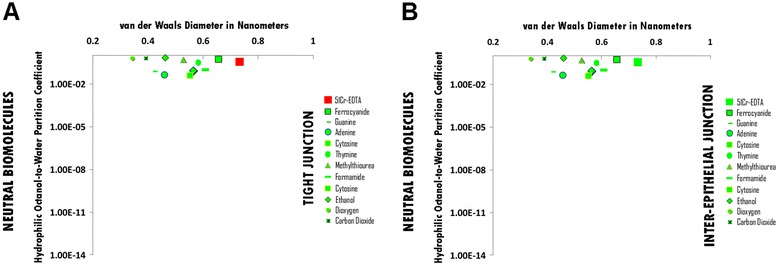
Panel **A**, Neutral Biomolecule Permeation across Tight Junction Pore Complexes ; Panel **B**, Neutral Biomolecule Permeation across Inter-Epithelial Pore Complexes. Hydrophilic Octanol-to-Water Partition Coefficient (Y-axis) and van der Waals Diameter in Nanometers; Green = Permeable; Red – Not Permeable.

**Figure 7:**
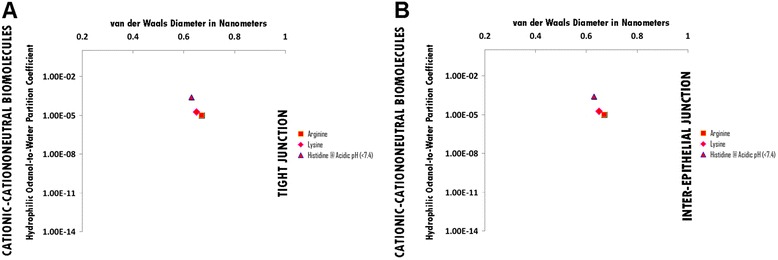
Panel **A**, Cationic- Cationoneutral Biomolecule Permeation across Tight Junction Pore Complexes ; Panel **B**, Cationic- Cationoneutral Biomolecule Permeation across Inter-Epithelial Pore Complexes. Hydrophilic Octanol-to-Water Partition Coefficient (Y-axis) and van der Waals Diameter in Nanometers; Green = Permeable; Red – Not Permeable.

**Figure 8:**
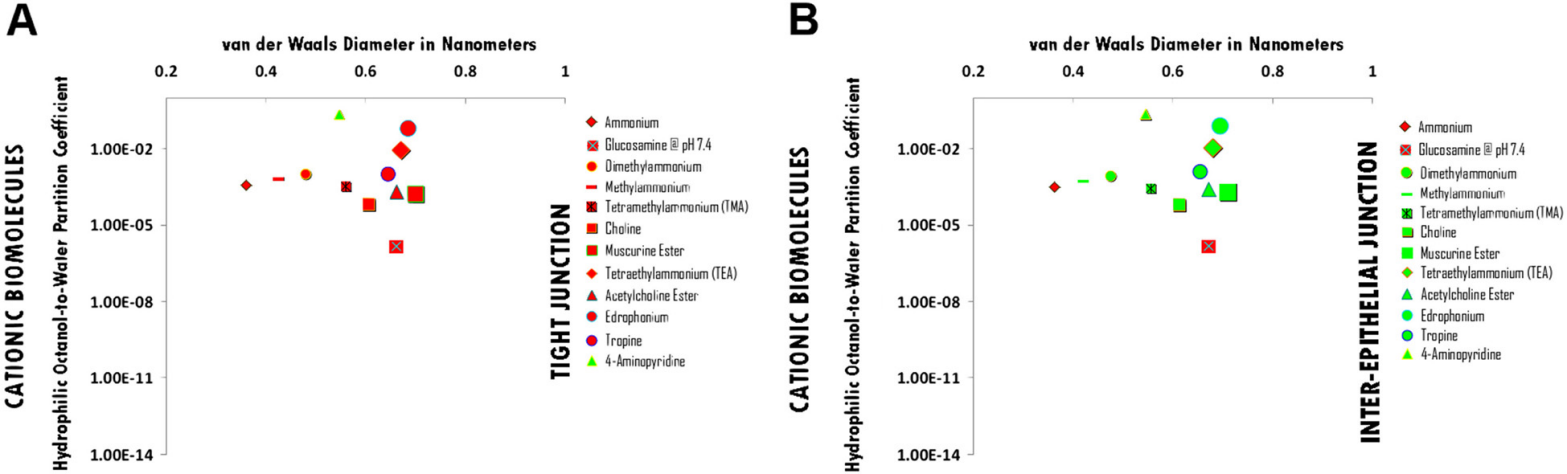
Panel **A**, Cationic Biomolecule Permeation across Tight Junction Pore Complexes ; Panel **B**, Cationic Biomolecule Permeation across Inter-Epithelial Pore Complexes. Hydrophilic Octanol-to-Water Partition Coefficient (Y-axis) and van der Waals Diameter in Nanometers; Green = Permeable; Red – Not Permeable.

**Figure 9:**
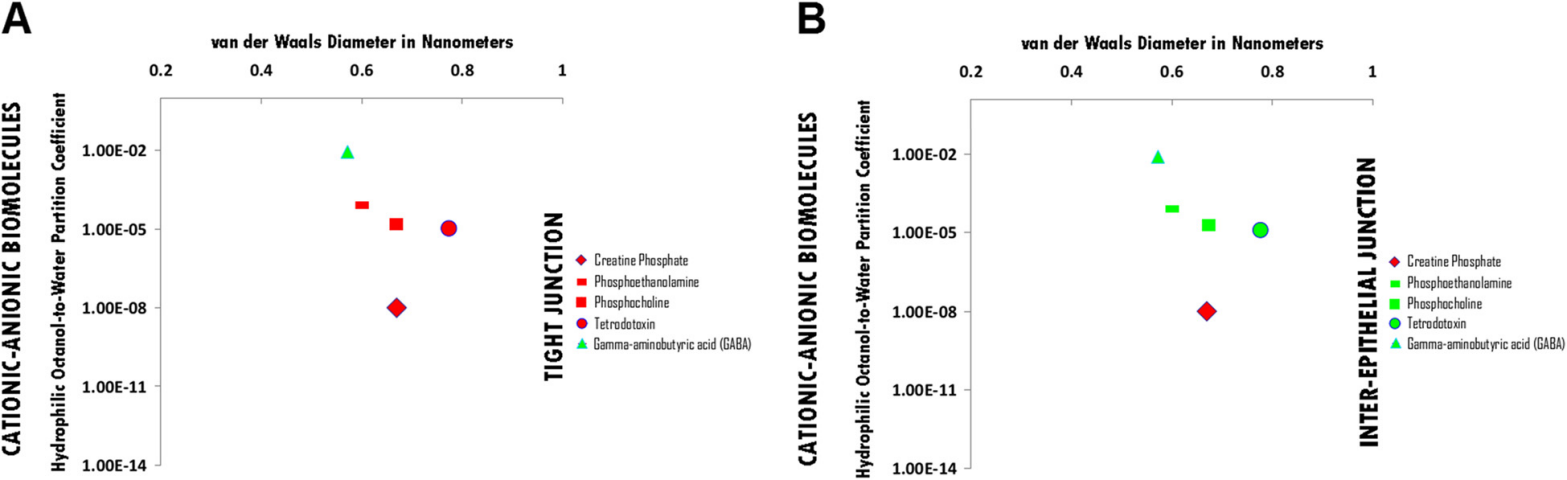
Panel **A**, Cationic-Anionic Biomolecule Permeation across Tight Junction Pore Complexes ; Panel **B**, Cationic-Anionic Biomolecule Permeation across Inter-Epithelial Pore Complexes. Hydrophilic Octanol-to-Water Partition Coefficient (Y-axis) and van der Waals Diameter in Nanometers; Green = Permeable; Red – Not Permeable.

The permeation potential of small molecule hydrophiles across aqueous pores of zona occludens tight junction complexes and inter-epithelial junction complexes is predictable upon mapping the predicted overall hydrophilicity octanol-to-water partition coefficient (HOWPC) [y-axis] versus the van der Waals Diameter (vdWD; nm) [x-axis] (Figures 1, 2, 3, 4, 5, 6, 7, 8 and 9: Panels A [Tight Junctions] and Panel B [Inter-epithelial Junctions]).

### Anionic hydrophile permeation thresholds across zona occludens tight junction pore complexes

The permeation threshold for anionic hydrophilic biomolecules across zona occludens tight junction pore complexes is between the hydrophilicity per molecular size ratio of Methyl(Flouro)phosphonate [Methyl(Flouro)phosphonic acid] (Log Dow: -2.85; vdWD: 0.50 nm; HOWPC-to-vdWD ratio: *-5.7 nm*^*-1*^) which is permeable at the lower aspect of the range, and that of Hydrogen Sulfate which does not chelate (Log Dow: -3.20; vdWD: 0.48 nm; HOWPC-to-vdWD ratio: *-6.7 nm*^*-1*^) which is not permeable at the upper aspect of the range. For the anionic hydrophile category across tight junction pore complexes, the HOWPC-to-vdWD ratio for a permeable hydrophile is *-5.7 nm*^*-1*^ at which the vdWD @ MAXimum HOWPC-to-vdWD is 0.50 nm (Figure 1, Panel A; Additional file 1: Table S1A. Hydrophiles: Anionic through Tight Junction Pore Complexes; Additional file 12: Table S12. Permeation Thresholds for Hydrophiles across Zona Occludens Tight Junction Pore Complexes).

The molecular size permeation threshold for anionic hydrophile permeation across zona occludens tight junction pore complexes is less than the molecular size of Mg-Citrate, which is not permeable with a vdWD of *0.63 nm*. For the anionic hydrophile category across zona occludens tight junction pore complexes, the vdWD @ MINimum HOWPC-to-vdWD is less than 0.63 nanometers (Figure 1, Panel A; Additional file 1: Table S1A. Hydrophiles: Anionic through Tight Junction Pore Complexes; Additional file 12: Table S12. Permeation Thresholds for Hydrophiles across Zona Occludens Tight Junction Pore Complexes).

### Anionic hydrophile permeation thresholds across inter-epithelial junction pore complexes

The permeation threshold for anionic hydrophilic biomolecules across inter-epithelial junction pore complexes is between the hydrophilicity per molecular size ratio of Carbonate (Carbonic Acid) (Log Dow: -3.80; vdWD: 0.45 nm; HOWPC-to-vdWD ratio: *-8.4 nm*^*-1*^) which is permeable at the lower aspect of the range, and that of Glucuronic Acid in the absence of chelation (Log Dow: -4.80; vdWD: 0.66 nm; HOWPC-to-vdWD ratio: *-8.5 nm*^*-1*^) which is not permeable at the upper aspect of the range. For the anionic hydrophile category across inter-epithelial junction pore complexes, the HOWPC-to-vdWD ratio for a permeable hydrophile is *-8.4 nm*^*-1*^ at which the vdWD @ MAXimum HOWPC-to-vdWD is 0.45 nm (Figure 1, Panel B; Additional file 1: Table S1B. Hydrophiles: Anionic through Inter-Epithelial Pore Complexes; Additional file 13: Table S13. Permeation Thresholds for Hydrophiles across Inter-Epithelial Pore Complexes).

The molecular size permeation threshold for anionic hydrophile permeation across inter-epithelial junction pore complexes is greater than the molecular size of Probenacid, which is permeable with a vdWD of *0.78 nm*. For the anionic hydrophile category across tight junction pore complexes, the vdWD @ MINimum HOWPC-to-vdWD is greater than 0.78 nanometers (Figure 1, Panel B; Additional file 1: Table S1B. Hydrophiles: Anionic through Inter-Epithelial Pore Complexes; Additional file 13: Table S13. Permeation Thresholds for Hydrophiles across Inter-Epithelial Pore Complexes).

### Anionic-cataniononeutral hydrophile permeation thresholds across zona occludens tight junction pore complexes

There is no permeation threshold for anionic-cataniononeutral hydrophilic biomolecules which do not permeate across zona occludens tight junction pore complexes due to un-opposed anionicity, while being similar in hydrophilicity per molecular size to the more hydrophilic for size anionic hydrophiles, while their molecular sizes are larger than those of the more hydrophilic anionic hydrophiles (Figure 2, Panel A; Additional file 2: Table S2A. Hydrophiles: Anionic-Cataniononeutral through Tight Junction Pore Complexes; Additional file 12: Table S12. Permeation Thresholds for Hydrophiles across Zona Occludens Tight Junction Pore Complexes).

### Anionic-cataniononeutral hydrophile permeation thresholds across inter-epithelial junction pore complexes

The permeation threshold for anionic-cataniononeutral hydrophilic biomolecules across inter-epithelial junction pore complexes is between the hydrophilicity per molecular size ratio of Kainate (Kainic Acid) (Log Dow: -5.00; vdWD: 0.71 nm; HOWPC-to-vdWD ratio: *-7.0 nm*^*-1*^) which is permeable at the lower aspect of the range, and that of N-Methyl-D-Asparatate (NMDA Acid) (Log Dow: -5.40; vdWD: 0.62 nm; HOWPC-to-vdWD ratio: *-8.7 nm*^*-1*^) which is not permeable at the upper aspect of the range. For the anionic-cataniononeutral hydrophile category across inter-epithelial junction pore complexes, the HOWPC-to-vdWD ratio for a permeable hydrophile is *-7.0 nm*^*-1*^ at which the vdWD @ MAXimum HOWPC-to-vdWD is 0.71 nm (Figure 2, Panel B; Additional file 2: Table S2B. Hydrophiles: Anionic-Cataniononeutral through Inter-Epithelial Pore Complexes; Additional file 13: Table S13. Permeation Thresholds for Hydrophiles across Inter-Epithelial Pore Complexes).

A molecular size permeation threshold does not exist for anionic-cataniononeutral hydrophiles across inter-epithelial junction pore complexes as un-opposed anionic hydrophilicity for size is the determinant for non-permeation (Figure 2, Panel B; Additional file 2: Table S2B. Hydrophiles: Anionic-Cataniononeutral through Inter-Epithelial Pore Complexes; Additional file 13: Table S13. Permeation Thresholds for Hydrophiles across Inter-Epithelial Pore Complexes).

### Pure polyneutral hydrophile permeation thresholds across zona occludens tight junction pore complexes

The permeation threshold for pure polyneutral hydrophilic biomolecules across zona occludens tight junction pore complexes is between the hydrophilicity per molecular size ratio of meso-Erythritol Sugar (Log Pow: -2.47; vdWD: 0.59 nm; HOWPC-to-vdWD ratio: *-4.2 nm*^*-1*^) which is permeable at the lower aspect of the range, and that of Glucose Sugar (Log Pow: -2.93; vdWD: 0.66 nm; HOWPC-to-vdWD ratio: *-4.5 nm*^*-1*^) which is not permeable at the upper aspect of the range. For the pure polyneutral hydrophile category across tight junction pore complexes, the HOWPC-to-vdWD ratio for a permeable hydrophile is *-4.2 nm*^*-1*^ at which the vdWD @ MAXimum HOWPC-to-vdWD is 0.59 nm (Figure 3, Panel A; Additional file 3: Table S3A. Hydrophiles: Pure Polyneutral through Tight Junction Pore Complexes; Additional file 12: Table S12. Permeation Thresholds for Hydrophiles across Zona Occludens Tight Junction Pore Complexes).

A molecular size permeation threshold is not applicable for pure polyneutral hydrophiles across tight junction pore complexes as hydrophilicity for size is the determinant for non-permeation which increases with increasing molecular size (Figure 3, Panel A; Additional file 3: Table S3A. Hydrophiles: Pure Polyneutral through Tight Junction Pore Complexes; Additional file 12: Table S12. Permeation Thresholds for Hydrophiles across Zona Occludens Tight Junction Pore Complexes).

### Pure polyneutral hydrophile permeation thresholds across inter-epithelial junction pore complexes

The permeation threshold for polyneutral hydrophilic biomolecules across inter-epithelial junction pore complexes is between the hydrophilicity per molecular size ratio of Sucrose Disaccharide Sugar (Log Pow: -4.53; vdWD: 0.81 nm; HOWPC-to-vdWD ratio:*-5.6 nm*^*-1*^) which is permeable at the lower aspect of the range, and that of Lactitol Disacchride Sugar (Log Pow: -5.50; vdWD: 0.82 nm; HOWPC-to-vdWD ratio:*-6.7 nm*^*-1*^) which is not permeable at the upper aspect of the range. For the polyneutral hydrophile category across inter-epithelial junction pore complexes, the HOWPC-to-vdWD ratio for a permeable hydrophile is *-5.6 nm*^*-1*^ at which the vdWD @ MAXimum HOWPC-to-vdWD is 0.81 nm (Figure 3, Panel B; Additional file 3: Table S3B. Hydrophiles: Pure Polyneutral through Inter-Epithelial Pore Complexes; Additional file 13: Table S13. Permeation Thresholds for Hydrophiles across Inter-Epithelial Pore Complexes).

A molecular size permeation threshold is not applicable for pure polyneutral hydrophiles across inter-epithelial junction pore complexes as hydrophilicity for molecular size is the determinant for non-permeation which increases with increasing molecular size (Figure 3, Panel B; Additional file 3: Table S3B. Hydrophiles: Pure Polyneutral through Inter-Epithelial Pore Complexes; Additional file 13: Table S13. Permeation Thresholds for Hydrophiles across Inter-Epithelial Pore Complexes).

### Neutral-Cataniononeutral and Cataniononeutral hydrophile permeation thresholds across zona occludens tight junction pore complexes

The permeation threshold for neutral-cataniononeutral and cataniononeutral hydrophilic biomolecules across zona occludens tight junction pore complexes is between the hydrophilicity per molecular size ratio of Alanine (Log Dow: -2.10; vdWD: 0.54 nm; HOWPC-to-vdWD ratio: *-3.9 nm*^*-1*^) which is permeable at the lower aspect of the range, and that of Threonine (Log Dow: -2.70; vdWD: 0.59 nm; HOWPC-to-vdWD ratio: *-4.6 nm*^*-1*^) which is not permeable at the upper aspect of the range. For the cataniononeutral hydrophile category across tight junction pore complexes, the HOWPC-to-vdWD ratio for a permeable hydrophile is *-3.9 nm*^*-1*^ at which the vdWD @ MAXimum HOWPC-to-vdWD is 0.54 nm (Figure 4, Panel A; Additional file 4: Table S4A. Hydrophiles: Neutral-Cataniononeutral and Cataniononeutral through Tight Junction Pore Complexes; Additional file 12: Table S12. Permeation Thresholds for Hydrophiles across Zona Occludens Tight Junction Pore Complexes).

The molecular size permeation threshold for cataniononeutral hydrophile permeation across zona occludens tight junction pore complexes is greater than that of Gabapentin, which is permeable with a vdWD of *0.69 nm*. For the cataniononeutral hydrophile category across tight junction pore complexes, the vdWD @ MINimum HOWPC-to-vdWD is greater than or equal to 0.69 nanometers (Figure 4, Panel A; Additional file 4: Table S4A. Hydrophiles: Neutral-Cataniononeutral and Cataniononeutral through Tight Junction Pore Complexes; Additional file 12: Table S12. Permeation Thresholds for Hydrophiles across Zona Occludens Tight Junction Pore Complexes).

### Neutral-Cataniononeutral and Cataniononeutral hydrophile permeation thresholds across inter-epithelial junction pore complexes

The permeation threshold for neutral-cataniononeutral and cataniononeutral hydrophilic biomolecules across inter-epithelial junction pore complexes is greater than the hydrophilicity per molecular size ratio of Asparagine @ pH < 7.4 (Log Dow -3.60; vdWD: 0.60 nm; HOWPC-to-vdWD ratio: *-6.0 nm*^*-1*^), which is permeable. For the neutral-cataniononeutral and cataniononeutral hydrophile category across inter-epithelial junction pore complexes, the HOWPC-to-vdWD ratio for a permeable hydrophile is greater than *-6.0 nm*^*-1*^ (Figure 4, Panel B; Additional file 4: Table S4B. Hydrophiles: Neutral-Cataniononeutral and Cataniononeutral through Inter-Epithelial Pore Complexes; Additional file 13: Table S13. Permeation Thresholds for Hydrophiles across Inter-Epithelial Pore Complexes).

The molecular size permeation threshold for neutral-cataniononeutral and cataniononeutral hydrophile across inter-epithelial junction pore complexes is greater than or equal to the molecular size of Gabapentin, which is permeable with a vdWD of *0.69* nm. For the cataniononeutral hydrophile category across inter-epithelial junction pore complexes, the vdWD @ MINimum HOWPC-to-vdWD is greater than or equal to 0.69 nanometers (Figure 4, Panel B; Additional file 4: Table S4B. Hydrophiles: Neutral-Cataniononeutral and Cataniononeutral through Inter-Epithelial Pore Complexes; Additional file 13: Table S13. Permeation Thresholds for Hydrophiles across Inter-Epithelial Pore Complexes).

### Mixed polyneutral hydrophile permeation thresholds across zona occludens tight junction pore complexes

A hydrophilicity per size threshold is not applicable for mixed polyneutral hydrophiles across zona occludens tight junction pore complexes as molecular size is the determinant for non-permeation (Figure 5, Panel A; Additional file 5: Table S5A. Hydrophiles: Mixed Polyneutral through Tight Junction Pore Complexes; Additional file 12: Table S12. Permeation Thresholds for Hydrophiles across Zona Occludens Tight Junction Pore Complexes).

The molecular size permeation threshold for polyneutral hydrophile permeation across zona occludens tight junction pore complexes is between the molecular size of Hydrogen Ascorbate Ester at pH 2, which is permeable with a vdWD of *0.64 nm*, and Thymidine, which is not permeable with a vdWD of *0.72 nm*. For the polyneutral hydrophile category across tight junction pore complexes, vdWD @ MINimum HOWPC-to-vdWD is less than 0.72 nanometers (Figure 5, Panel A; Additional file 5: Table S5A. Hydrophiles: Mixed Polyneutral through Tight Junction Pore Complexes; Additional file 12: Table S12. Permeation Thresholds for Hydrophiles across Zona Occludens Tight Junction Pore Complexes).

### Mixed polyneutral hydrophile permeation thresholds across inter-epithelial junction pore complexes

The permeation threshold for mixed polyneutral hydrophilic biomolecules across inter-epithelial junction pore complexes is greater than the hydrophilicity per molecular size ratio of Glucosamine at basic pH (Log Pow: -3.1; vdWD: 0.66 nm; HOWPC-to-vdWD ratio:*-4.7 nm*^*-1*^) which is permeable. For the mixed polyneutral hydrophile category across inter-epithelial junction pore complexes, the HOWPC-to-vdWD ratio for a permeable hydrophile is greater than *-4.7 nm*^*-1*^ (Figure 5, Panel B; Additional file 5: Table S5B. Hydrophiles: Mixed Polyneutral through Inter-Epithelial Pore Complexes; Additional file 13: Table S13. Permeation Thresholds for Hydrophiles across Inter-Epithelial Pore Complexes).

The molecular size permeation threshold for mixed polyneutral hydrophile permeation across inter-epithelial junction pore complexes is greater than the molecular size of Adenosine, which is permeable with a vdWD of *0.74 nm*. For the mixed polyneutral hydrophile category across inter-epithelial junction pore complexes, the vdWD @ MINimum HOWPC-to-vdWD is greater than 0.74 nanometers (Figure 5, Panel B; Additional file 5: Table S5B. Hydrophiles: Mixed Polyneutral through Inter-Epithelial Pore Complexes; Additional file 13: Table S13. Permeation Thresholds for Hydrophiles across Inter-Epithelial Pore Complexes).

### Neutral hydrophile permeation thresholds across zona occludens tight junction pore complexes

The permeation threshold for neutral hydrophilic biomolecules across zona occludens tight junction pore complexes is greater than the hydrophilicity per molecular size ratio of Urea (Log Pow: -1.36; vdWD: 0.46 nm; HOWPC-to-vdWD ratio: *-3.0 nm*^*-1*^) which is permeable. For the neutral hydrophile category across tight junction pore complexes, the HOWPC-to-vdWD ratio for a permeable hydrophile is greater than *-3.0 nm*^*-1*^ (Figure 6, Panel A; Additional file 6: Table S6A. Hydrophiles: Neutral through Tight Junction Pore Complexes; Additional file 12: Table S12. Permeation Thresholds for Hydrophiles across Zona Occludens Tight Junction Pore Complexes).

The molecular size permeation threshold for neutral hydrophile permeation across zona occludens tight junction pore complexes is between that of Ferrocyanide, which is permeable with a vdWD of *0.66 nm*, and the molecular size of 51Cr-EDTA, which is not permeable with a vdWD of *0.73 nm*. For the neutral hydrophile category across tight junction pore complexes, the vdWD @ MINimum HOWPC-to-vdWD is between 0.66 nanometers and 0.73 nanometers (Figure 6, Panel A; Additional file 6: Table S6A. Hydrophiles: Neutral through Tight Junction Pore Complexes; Additional file 12: Table S12. Permeation Thresholds for Hydrophiles across Zona Occludens Tight Junction Pore Complexes).

### Neutral hydrophile permeation thresholds across inter-epithelial junction pore complexes

The permeation threshold for neutral hydrophilic biomolecules across inter-epithelial junction pore complexes is greater than the hydrophilicity per molecular size ratio of Urea (Log Pow: -1.36; vdWD: 0.46 nm; HOWPC-to-vdWD ratio: *-3.0 nm*^*-1*^) which is permeable. For the neutral hydrophile category across inter-epithelial junction pore complexes, the HOWPC-to-vdWD ratio is greater than *-3.0 nm*^*-1*^ (Figure 6, Panel B; Additional file 6: Table S6B. Hydrophiles: Neutral through Inter-Epithelial Pore Complexes; Additional file 13: Table S13. Permeation Thresholds for Hydrophiles across Inter-Epithelial Pore Complexes).

The molecular size permeation threshold for neutral hydrophile permeation across inter-epithelial junction pore complexes is greater than the molecular size of 51Cr-EDTA, which is permeable with a vdWD of *0.73 nm*. For the neutral hydrophile category across inter-epithelial junction pore complexes, the vdWD @ MINimum HOWPC-to-vdWD is greater than or equal to 0.73 nanometers (Figure 6, Panel B; Additional file 6: Table S6B. Hydrophiles: Neutral through Inter-Epithelial Pore Complexes; Additional file 13: Table S13. Permeation Thresholds for Hydrophiles across Inter-Epithelial Pore Complexes) and equal to atleast 0.81 nanometers (Additional file 13: Table S13. Permeation Thresholds for Hydrophiles across Inter-Epithelial Pore Complexes).

### Cationic-Cataniononeutral hydrophile permeation thresholds across zona occludens tight junction pore complexes versus inter-epithelial junction pore complexes

There is no permeation threshold for cationic-cataniononeutral hydrophilic biomolecules which do not permeate across zona occludens tight junction pore complexes or across inter-epithelial junction pore complexes due to the presence of unopposed cationicity while being similar in hydrophilicity per molecular size to the more hydrophilic for size cationic-anionic hydrophiles (Figure 7, Panels A and B; Additional file 7: Table S7A. Hydrophiles: Cationic-Cataniononeutral through Tight Junction Pores Complexes; Additional file 7: Table S7B. Hydrophiles: Cationic-Cataniononeutral through Inter-Epithelial Pore Complexes; Additional file 12: Table S12. Permeation Thresholds for Hydrophiles across Zona Occludens Tight Junction Pore Complexes; Additional file 13: Table S13. Permeation Thresholds for Hydrophiles across Inter-Epithelial Pore Complexes).

### Cationic hydrophile permeation thresholds across zona occludens tight junction pore complexes

The permeation threshold for cationic hydrophilic biomolecules across zona occludens tight junction pore complexes is between the hydrophilic per molecular size ratio of 4-Aminopyridine (Log Dow: -0.57; vdWD: 0.55 nm; HOWPC-to-vdWD ratio: *-1.0 nm*^*-1*^) which is permeable at the lower aspect of the range, and that of Edrophonium (Log Dow: -1.2; vdWD: 0.69 nm; HOWPC-to-vdWD ratio: *-1.8 nm*^*-1*^) which is not permeable at the upper aspect of the range. For the cationic hydrophile category across tight junction pore complexes, the HOWPC-to-vdWD ratio for a permeable hydrophile is *-1.0 nm*^*-1*^ at which the vdWD @ MAXimum HOWPC-to-vdWD is 0.55 nm (Figure 8, Panel A; Additional file 8: Table S8A. Hydrophiles: Cationic through Tight Junction Pore Complexes; Additional file 12: Table S12. Permeation Thresholds for Hydrophiles across Zona Occludens Tight Junction Pore Complexes).

A molecular size permeation threshold does not exist for cationic hydrophiles across zona occludens tight junction pore complexes as unopposed cationic hydrophilicity for size is the determinant for non-permeation (Figure 8, Panel A; Additional file 8: Table S8A. Hydrophiles: Cationic through Tight Junction Pore Complexes; Additional file 12: Table S12. Permeation Thresholds for Hydrophiles across Zona Occludens Tight Junction Pore Complexes).

### Cationic hydrophile permeation thresholds across inter-epithelial junction pore complexes

The permeation threshold for cationic hydrophilic biomolecules across inter-epithelial junction pore complexes is between the hydrophilicity per molecular size ratio of Methylammonium (Log Dow: -3.2; vdWD: 0.43 nm; HOWPC-to-vdWD ratio: *-7.5 nm*^*-1*^) which is permeable at the lower aspect of the range, and that of Glucosamine (Log Dow: -5.75 ; vdWD: 0.66 nm; HOWPC-to-vdWD ratio: *-8.7 nm*^*-1*^) which is not permeable at the upper aspect of the range. For the cationic hydrophile category across inter-epithelial junction pore complexes, the HOWPC-to-vdWD ratio for a permeable hydrophile is *-7.5 nm*^*-1*^ at which the vdWD @ MAXimum HOWPC-to-vdWD is 0.43 nm (Figure 8, Panel B; Additional file 8: Table S8B. Hydrophiles: Cationic through Inter-Epithelial Pore Complexes; Additional file 13: Table S13. Permeation Thresholds for Hydrophiles across Inter-Epithelial Pore Complexes).

The molecular size permeation threshold for cationic hydrophile permeation across inter-epithelial junctional complex pores is greater than or equal to the molecular size of Muscurine Ester (Muscurine), which is permeable with a vdWD of *0.70 nm*. For the cationic hydrophile category across inter-epithelial junction pore complexes, the vdWD @ MAXimum HOWPC-to-vdWD is greater than or equal to 0.70 nanometers (Figure 8, Panel B; Additional file 8: Table S8B. Hydrophiles: Cationic through Inter-Epithelial Pore Complexes; Additional file 13: Table S13. Permeation Thresholds for Hydrophiles across Inter-Epithelial Pore Complexes).

### Cationic-Anionic hydrophile permeation thresholds across zona occludens tight junction pore complexes

The permeation threshold for cationic-anionic hydrophilic biomolecules across zona occludens tight junction pore complexes is between the hydrophilicity per molecular size ratio of gamma-aminobutyric acid (GABA) (Log Dow: -2.1; vdWD: 0.57 nm; HOWPC-to-vdWD ratio: *-3.7 nm*^*-1*^) which is permeable at the lower aspect of the range, and that of Tetrodotoxin (TTX) (Log Dow: -4.90; vdWD: 0.77 nm; HOWPC-to-vdWD ratio: -*6.3 nm*^*-1*^) which is not permeable at the upper aspect of the range. For the cationic-anionic hydrophile category across tight junction pore complexes, the HOWPC-to-vdWD ratio for a permeable hydrophile is *-3.7 nm*^*-1*^ at which the vdWD @ MAXimum HOWPC-to-vdWD is 0.57 nm (Figure 9, Panel A; Additional file 9: Table S9A. Hydrophiles: Cationic-Anionic through Tight Junction Pore Complexes; Additional file 12: Table S12. Permeation Thresholds for Hydrophiles across Zona Occludens Tight Junction Pore Complexes).

A molecular size permeation threshold does not exist for cationic-anionic hydrophiles across zona occludens tight junction pore complexes as hydrophilicity for molecular size is the determinant for non-permeation irrespective of size (Figure 9, Panel A; Additional file 9: Table S9A. Hydrophiles: Cationic-Anionic through Tight Junction Pore Complexes; Additional file 12: Table S12. Permeation Thresholds for Hydrophiles across Zona Occludens Tight Junction Pore Complexes).

### Cationic-Anionic hydrophile permeation thresholds across inter-epithelial junction pore complexes

The permeation threshold for cationic-anionic hydrophilic biomolecules across inter-epithelial junction pore complexes is between the hydrophilicity per molecular size ratio of Phosphocholine (Log Dow: -4.80; vdWD: 0.67 nm; HOWPC-to-vdWD ratio: -*7.2 nm*^*-1*^) which is permeable at the lower aspect of the range, and that of Creatine Phosphate (Log Dow -8.00; vdWD: 0.67 nm; HOWPC-to-vdWD ratio: *-12..0 nm*^*-1*^) which is not permeable at the upper aspect of the range. For the cationic-anionic hydrophile category across inter-epithelial junction pore complexes, the HOWPC-to-vdWD ratio for a permeable hydrophile is *-7.2 nm*^*-1*^ at which the vdWD @ MAXimum HOWPC-to-vdWD is 0.67 nm (Figure 9, Panel B; Additional file 9: Table S9B. Hydrophiles: Cationic-Anionic through Inter-Epithelial Pore Complexes; Additional file 13: Table S13. Permeation Thresholds for Hydrophiles across Inter-Epithelial Pore Complexes).

The molecular size permeation threshold for cationic-anionic hydrophile permeation across inter-epithelial junction pore complexes is greater than or equal to the molecular size of Tetrodotoxin, which is permeable with a vdWD of *0.77 nm*. For the cationic-anionic hydrophile category across inter-epithelial junction pore complexes, the vdWD @ MAXimum HOWPC-to-vdWD is greater than or equal to 0.77 nanometers (Figure 9, Panel B; Additional file 9: Table S9B. Hydrophiles: Cationic-Anionic through Inter-Epithelial Pore Complexes; Additional file 13: Table S13. Permeation Thresholds for Hydrophiles across Inter-Epithelial Pore Complexes).

### Endogenous and non-endogenous non-metal and metal permeation thresholds across zona occludens tight junction pore complexes

The permeation threshold of cationic non-metals and metals across zona occludens tight junction pore complexes is between the cationicity per size ratio of Potassium (K+; Cationization-to-Atomic Diameter ratio: +2.20 nm^-1^) and that of Sodium (Na+; Cationization-to-Atomic Diameter ratio: + 2.69 *nm*^*-1*^), where K+ and CH3-Hg+ (Cationization-to-Diameter Ratio: +2.36 nm^-1^) are permeable across zona occludens tight junction pore complexes, whereas Na+ is not (Additional file 10: Table S10A. Endogenous and Non-endogenous Non-Metals and Metals through Zona Occludens Tight Junction Pore Complexes).

### Endogenous and non-endogenous non-metal and metal permeation thresholds across inter-epithelial junction pore complexes

The permeation threshold of cationic non-metals and metals across inter-epithelial junction pore complexes is at about the cationicity per size ratio of Calcium (Ca2+; Cationization-to-Atomic Diameter ratio: +5.08 nm^-1^). The cationicity per size ratio of Lead (Pb2+; Cationization-to-Atomic Diameter ratio:+5.71 nm^-1^), where Na+ (Cationization-to-Atomic Diameter ratio: +2.69 nm^-*1*^), and CH3-Hg+ (Cationization-to-Atomic Diameter ratio: + 2.36 *nm*^*-1*^) are fully permeable across inter-epithelial junction pore complexes, whereas Calcium (Ca2+) is partially permeable, while lead (Pb2+) and Magnesium (Mg2+; Cationization-to-Diameter ratio: +6.25 nm^-1^) are not permeable across inter-epithelial junction pore complexes (Additional file 10: Table S10B. Endogenous and Non-endogenous Non-Metals and Metals through Inter-Epithelial Pore Complexes).

### Biological Halogen permeation thresholds across zona occludens tight junction pore complexes versus inter-epithelial junction pore complexes

The permeation threshold of biological halogens across zona occludens tight junction pores complexes is between the anionicity per size ratio of Chlorine (Cl-; Anionization-to-Atomic Diameter Ratio: -4.80 nm^-1^) and Bromine (Br-; Anionization-to-Atomic Diameter Ratio:- 4.17 nm^-1^), which is the anionicity for size ratio cut-off range for cell membrane permeation where Iodine (I-; Anionization-to-Atomic Diameter Ratio: - 3..57 nm^-1^) and Bromine are also permeable across cell membranes (Additional file 11: Table S11A. Biological Halogens through Zona Occludens Tight Junction Pore Complexes).

The permeation threshold of biological halogens across inter-epithelial junction pore complexes is between the anionicity per size ratio of Fluorine (F-; Anionization-to-Atomic Diameter Ratio: - 7.81 nm^-1^) and that of Chlorine (Cl-; Anionization-to-Atomic Diameter Ratio: - 4.80 nm^-1^), where Fluorine is not permeable across inter-epithelial junction pore complexes, while Chlorine, Bromine and Iodine are all permeable across inter-epithelial junction pore complexes (Additional file 11: Table S11B. Biological Halogens through Inter-Epithelial Pore Complexes).

## Discussion

### Permeation thresholds of anionic and anionic-cataniononeutral small molecule hydrophiles across endothelial and epithelial junctional pore complexes

Anionic small molecule hydrophiles are carboxylic, phosphoric, ascorbic and sulfonic acids, and anionic-cataniononeutral small molecule hydrophiles are amino acids with a carboxylic or phosphoric acid with sufficient (S) negative charge separation in molecular space in addition to a positively charged amine insufficiently separated (IS) from a negatively charged carboxylic acid in molecular space.

In the case of anionic small molecule hydrophiles, which become too hydrophilic for size to permeate across junction pore complexes at certain thresholds, the hydrophilicity for size permeation threshold across tight junction pore complexes is between a HOWPC-to-vdWD ratio of -5.7 and -6.7 *nm*^*-1*^ at which the size for permeation is at a nadir of 0.50 nanometers, and the hydrophilicity for size permeation threshold across inter-epithelial junction pore complexes is between a HOWPC-to-vdWD ratio of -8.4 and -8.5 *nm*^*-1*^ at which the size for permeation is at a nadir of 0.48 nanometers; whereas, in the case of anionic-cataniononeutral hydrophiles, these are not permeable across tight junction pore complexes due to un-opposed anionic charge exclusion, and the hydrophilicity for size permeation threshold across inter-epithelial junction pore complexes, the hydrophilicity for size permeation threshold is between a HOWPC-to-vdWD ratio of -7.0 and -8.7 *nm*^*-1*^ at which the size for permeation is at a nadir of 0.71 nanometers.

These findings of this study are consistent with the supposition that anionic and anionic-catanioneutral small molecule hydrophile hydrophilicity is an important determinant of barrier permeation potential, whereby those with sufficient un-opposed anionic charge hydrophilicity are excluded from junction pores; in contrast, in the case of small molecule hydrophiles of less significant anionic hydrophilicity, these hydrophiles are permeable at larger molecular sizes, for example, in the case of the least hydrophilic anionic hydrophile Probenacid with a HOWPC-to-vdWD ratio of -1.1 *nm*^*-1*^ that is permeable across inter-epithelial junction complexes at a molecular size of 0.78 nanometers, whereby its larger molecular size would be the reason for its saturable absorption kinetics (Selen et al. 1982).

With regards to anionic small molecule hydrophiles that are hydrophilicity-restricted from permeation across junction pore complexes, only the subset of these hydrophiles with the ability to become less hydrophilic can permeate across the gastrointestinal inter-epithelial pore complexes, either via the chelation of divalent or trivalent cations, which, for example, is the case of citrate (-16.6 *nm*^*-1*^) as Mg2+-citrate (-2.1 *nm*^*-1*^) (Lindberg et al. 1990) and Foscarnet (-16.8 *nm*^***-1***^) as Mg2+-Foscarnet (-1.6 *nm*^*-1*^) (Noormohamed et al. 1998) upon neutralization of anionic hydrophilicity, or via metabolic breakdown, which, for example, is the case of sulfate upon bacterial metabolism into hydrogen sulphide (H2S) gas (Florin et al. 1991). This neutralization of hydrophilicity or metabolism to less hydrophilic forms the basis for their ability to permeate across inter-epithelial junction pore complexes, and thereafter, freely across mesenteric diaphragm fenestrated blood capillaries into systemic blood circulation without restriction.

Based on these observations, it is ascertained that impermeable anionic hydrophiles are entirely compartmentally biosynthesized in the biological system *in vivo*, either via oxidation of hydrogen sulphide in the case of sulphate (HOWPC-to-vdWD ratio: -11.5 *nm*^*-1*^; vdWD: 0.48 nm), or carboxylation of sugars in the case of sialyate (sialic acid) (HOWPC-to-vdWD ratio: -9.4 *nm*^*-1*^; vdWD: 0.79 nm) and glucuronate (glucuronic acid) (HOWPC-to-vdWD ratio: -8.5 *nm*^*-1*^; vdWD 0.66 nm), which would stand to be extracellular for the sulphates, sialyates and glucuronates (hyaluronates (Janmey et al. 2014)) that become apart of the endothelial and epithelial glycocalyces (Squire et al. 2001; Reitsma et al. 2007; Henry and Duling 1999; Martins and Bairos 2002), and apart of the extracellular matrixes in tissue-organs with arteriolarly supplied less restrictive fenestrated and continuous blood capillaries, for example, the kidney glomeruli extracellular matrix, and to a lesser degree, the cardiac and skeletal muscle extracellular matrix. Therefore, it can be deduced that the primary mechanism underlying differences in the thicknesses of the endothelial glycocalyical coats of different tissue capillary beds (Brouland et al. 1999; Nieuwdorp et al. 2008) are the relative differences in capillary wall endothelium pore sizes cum relative exclusion of anionic hydrophiles within the -8.5 to -11.5 *nm*^*-1*^ range, which is the optimal hydrophilicity for size range for the formation of a thick glycocalyx layer for more restrictive barriers including for the epithelial barrier (Martins and Bairos 2002). It is further notable that anionic hydrophiles most hydrophilic for size tend to remain locally within the interstitia of tissue spaces where they are secreted, even in close proximity to fenestrated blood capillaries, which is the case of the interstitial matrix of bone where pyrophosphoric acid remains at alkaline pH, as at basic pH it has a HOWPC-to-vdWD ratio of 20.2 *nm*^*-1*^ with an anionicity of -4, whereby it is able to remain and associate closely with permeable divalent calcium (Ca2+) cations to form mineral hydroxyapatite upon pseudochelation of multiple Ca2+ cations.

### Permeation thresholds of pure polyneutral, neutral-cataniononeutral, mixed polyneutral and neutral small molecule hydrophiles across endothelial and epithelial junctional pore complexes

Pure polyneutral small molecule hydrophiles are sugar biomolecules which are polyhydroxylated; neutral-cataniononeutral small molecule hydrophiles are amino acids with a neutral group in addition to a positively charged amine insufficiently separated (IS) from a negatively charged carboxylic acid in molecular space; mixed polyneutral small molecule hydrophiles are neutral sugars with neutral aminated groups; and neutral small molecule hydrophiles are neutral molecules irrespective of atom constitution, categorically in order of most hydrophilic for molecular size to least.

In the case of pure polyneutral hydrophiles, there is a direct relationship between increasing molecular size and increasing hydrophilicity with increasing hydroxylation, whereby hydrophilicity for molecular size becomes the primary determinant for diffusion restriction to permeation across junctional complexes: Pure polyneutral hydrophiles become too hydrophilic for size to permeate across tight junction pore complexes in between a HOWPC-to-vdWD ratio of -4.2 (meso-erythritol) and -4.5 *nm*^*-*1^ (glucose), and across inter-epithelial junction pore complexes in between a HOWPC-to-vdWD ratio of -5.6 (sucrose) and -6.7 *nm*^*-1*^ (lactitol). In contrast to pure polyneutral small molecule hydrophiles, in the case of neutral-cataniononeutral, mixed polyneutral and neutral small molecule hydrophiles, these are categorically less hydrophilic for size, with neutral-cataniononeutral hydrophiles being greater in hydrophilicity for size due to presence of with insufficiently separated (IS) positive and negative charge over molecular space than the mixed polyneutral hydrophiles. Therefore, in the case of neutral-cataniononeutral hydrophiles, hydrophilicity for size becomes the primary determinant for permeation restriction across inter-endothelial and inter-epithelial junction complexes: Neutral-cataniononeutral small molecule hydrophiles become too hydrophilic for size to permeate across tight junction pore complexes in between a HOWPC-to-vdWD ratio of -3.9 (Alanine) and -4.6 *nm*^*-*1^ (Threonine) at which the size for permeation is at a nadir of 0.54 nanometers, however, neutral-cataniononeutral hydrophiles are not restricted to permeation across the inter-epithelial junction pore complexes at atleast a HOWPC-to-vdWD ratio of -6.0 *nm*^*-*1^ (Asparagine). In contrast to pure polyneutral and neutral-cataniononeutral hydrophiles, for mixed polyneutral and neutral small molecule hydrophiles the primary determinant of restriction to permeation is molecular size, which become too large to permeate across tight junction pore complexes in between a vdWD of 0.66 and 0.73 nm, however, mixed polyneutral and neutral hydrophiles are not restricted to permeation across the inter-epithelial junction pore complexes of vdWDs of atleast 0.74 nm and would be permeable at vdWDs of atleast 0.81 nm as pure polyneutral hydrophiles are permeable across inter-epithelial junction pore complexes at a at vdWD of 0.81 nanometers.

This being the case, correlations can be established for the permeation potential of these uncharged small molecule hydrophiles on their conserved biophysical properties of hydrophilicity versus molecular size in context of physiologic study data findings on relative differences in the permeation of a known subset of the small molecule hydrophiles including raffinose (Fenstermacher and Johnson 1966), lactitol (Roberts 1975), sucrose (Evans et al. 1974; Hewetson et al. 2006), glucose (Davson 1955), 51Cr-ETDA (Sorensen 1974), ferrocyanide (Seiguer and Mancini 1971) and urea (Fenstermacher and Johnson 1966) across the capillary wall blood-brain barrier (BBB) (Fenstermacher and Johnson 1966; Davson and Welch 1971; Sorensen 1974; Evans et al. 1974) and the epithelial barriers, the blood-ventricular cerebrospinal fluid barrier (BVCSFB) (Fenstermacher and Johnson 1966; Davson and Welch 1971; Sorensen 1974; Evans et al. 1974) and the fluid-intestinal barrier (FIB) (Roberts 1975; Hewetson et al. 2006). These deductive correlations on the relative contributions of the more restrictive capillary wall BBB and the less restrictive BVCSFB to small molecule hydrophile permeation can be further refined on the basis of knowledge that both the BBB and the BVCSFB contribute to permeation into the brain parenchyma during the natal period, whereas only the BVCSFB contributes to permeation into the brain parenchyma following the progressive developmental closure of the BBB during the natal period. Therefore, *in vivo* physiologic studies in which the permeation of systemically administered polyneutral and neutral small molecule hydrophiles into the central nervous system (CNS) has been studied offer valuable insight into the potential for small molecule hydrophile permeation across the BBB (Fenstermacher and Johnson 1966) as compared to that across the BVCSFB (Evans et al. 1974). Based on the findings of these studies taken altogether (Fenstermacher and Johnson 1966; Evans et al. 1974), it can be appreciated that there is a significant decrease in the influx of sucrose into the brain parenchyma over the course of fetal development due to the progressive closure of the BBB during the natal period, after which the BVCSFB remains the pathway for the entry of disaccharide sucrose into the CNS across the inter-epithelial junction pore complexes of the choroid plexus epithelium that constitute the BVCSFB, which is by the way of the ultrafiltration of blood plasma through the BVCSFB as CSF. In contrast to sucrose (HOWPC-to-vdWD ratio: -5.6 *nm*^***-1***^; vdWD: 0.81 nm), which is permeable across epithelial barriers such as the BVCSFB and the fluid-intestinal barrier (Evans et al. 1974; Hewetson et al. 2006), lactitol is not permeable (Roberts 1975), and based on the findings of this study, the impermeability of lactitol (HOWPC-to-vdWD ratio: -6.7 *nm*^***-1***^; vdWD 0.82 nm) to inter-epithelial junctional complexes is attributable to lactitol’s greater hydrophilicity for molecular size, the molecular sizes of sucrose and lactitol being similar.

As to the permeation potential of pure polyneutral hydrophiles less hydrophilic for size across the BBB as compared to the BVCSFB, it is a particular *in vivo* physiologic study that offers insight into the relative restrictiveness of these barriers to the permeation of monosaccharide glucose (HOWPC-to-vdWD ratio: -4.5 *nm*^*-1*^; vdWD: 0.66 nm) into the CNS parenchyma, which is related to the amount of hydrophilicity for size. In this study (Davson 1955), the permeation of intraperitoneally administered small molecule hydrophiles, cations and anions across the BVCSFB into the CSF was studied in comparison to permeation across the blood-choriocapillaris aqueous humor-barrier (BCAHB) into the aqueous humor, in which it was determined (Table one of Reference Davson (1955)) that the cell membrane- and BBB-impermeant cation, Na+, achieves equivalent concentrations in the acellular aqueous humor across the diaphragm fenestrated blood capillaries of the choriocapillaris as well as in the CSF across the diaphragm fenestrated blood capillaries of the cellular choroid plexus and then the epithelial BVCSFB in series, as compared to the cell membrane- and BBB-permeant cation, K+, which achieves equivalent concentration to Na+ in the acellular aqueous humor but only ~50% of the concentration of Na+ in the CSF, through the cellular choroid plexus; in the case of cell membrane restricted, but permeant, polyneutral hydrophile, glucose, it achieves ~90% of the concentration of Na+ in the relatively acellular non-metabolic milieu of the aqueous humor as compared to approximately 60% of the concentration of Na+ in the CSF across the BVCSFB, which is through the relatively cellular metabolic milieu of the CNS choroid plexus: This observation is consistent with the intra-cellular metabolism of a greater proportion of the glucose (~30%) in the choroid plexus, with the extracellular glucose proportion only being permeant across the BVCSFB for accumulation into the CSF (~60%), whereby this observation taken in context of the fact that the reflection coefficient of glucose to the combination of the BBB and BVCSFB barriers of the CNS is 0.89 (Fenstermacher and Johnson 1966), and close to unity, implies that the BVCSFB is the pathway by which glucose enters the CNS parenchyma rather than the BBB via its equilibration with in the ventricular CSF first, and then, equilibration within the CNS parenchyma itself.

The molecular size constraints to hydrophile permeation across restrictive barriers as modeled *in silico* herein can be appreciated by deductive correlations with *in vivo* physiologic studies on the permeability of 51Cr-EDTA (HOWPC-to-vdWD ratio: -0.6 *nm*^*-1*^; vdWD: 0.73 nm) across the inter-epithelial junction complexes of the BVCSFB in the CNS parenchymal interstitium (Sorensen 1974) and on the permeability of Ferrocyanide (HOWPC-to-vdWD ratio: -0.4 *nm*^*-1*^; vdWD: 0.66 nm) across the inter-endothelial tight junction pore complexes of the blood-testes-barrier (BTESTB) capillary walls into the testes parenchymal interstitium (Seiguer and Mancini 1971; Holash et al. 1993). Based on the findings of the former Sorensen (1974), intravenously administered 51Cr-EDTA (HOWPC-to-vdWD ratio: -0.6 *nm*^*-1*^; vdWD: 0.73 nm) is permeable across the inter-epithelial junction complexes of the BVCSFB into the CNS parenchyma, implying that 51Cr-EDTA is permeable across the inter-epithelial junction complexes of the BVCSFB (Sorensen 1974), as well as the FIB (Soderholm et al. 2002), but not the across the inter-endothelial tight junction pore complexes; and based on findings of the later Seiguer and Mancini (1971), intravenously administered Ferrocyanide (HOWPC-to-vdWD ratio: -0.4 *nm*^*-1*^; vdWD: 0.66 nm) is permeable across the across the inter-endothelial tight junction pore complexes of the BTESTB capillary walls into the testes parenchyma.

By taking into consideration the observations of this study on the permeation potential of the spectrum of small molecule hydrophiles of neutralized biophysical character, ranging from the pure polyneutral, neutral-cataniononeutral, mixed polyneutral to neutral, it can be surmised that only the subset of these neutralized hydrophiles that are either absolutely or relatively restricted to permeation across inter-endothelial tight junction or inter-epithelial junction pore complexes due to sufficient hydrophilicity for size or due to sufficient molecular size (pure polyneutral, neutral-cataniononeutral, mixed polyneutral), become local substrates for either endothelial or epithelial cell uptake, which is via cell membrane protein channel for local intracellular uptake, and furthermore, for junction pore complex permeable neutralized hydrophiles, the interplay of hydrophilicity per size and absolute molecular size are the determinants of saturable kinetics of the second order, for example, as it applies to the trans-barrier pore permeation kinetics of essential amino acids (Aoyagi et al. 1988; Pardridge 1977).

### Permeation thresholds of cationic-cataniononeutral, cationic and cationic-anionic small molecule hydrophiles across endothelial and epithelial junctional pore complexes

Cationic-cataniononeutral small molecule hydrophiles are amino acids with a charged amine with sufficient (S) positive charge separation in molecular space in addition to a positively charged amine insufficiently separated (IS) from a negatively charged carboxylic acid in molecular space; cationic small molecule hydrophiles consist of a singularly charged amine; and cationic-anionic small molecule hydrophiles consist of a charged amine and phosphoric and/or carboxylic acid with partial sufficient (PS) separation of opposing charges in molecular space.

Cationic-cataniononeutral hydrophiles are charge-excluded to junctional pore complexes and precluded from permeation across both endothelial and epithelial barriers due to the presence of un-opposed cationic charge in the setting of additional molecular charge which results in enough hydrophilicity for size: This, for example, is the case for Arginine (HOWPC-to-vdWD ratio: -7.5 *nm*^*-1*^; vdWD: 0.67 nm), Lysine (HOWPC-to-vdWD ratio: -7.3 *nm*^*-1*^; vdWD: 0.65 nm) and Histidine at acidic pH (HOWPC-to-vdWD ratio: -5.7 *nm*^*-1*^; vdWD: 0.63 nm). Therefore, this finding suggests that, for permeation restricted cationic-cataniononeutral hydrophiles, the un-opposed cationicity interacts with cell membrane surfaces resulting in cellular contraction, inter-cellular separation and in the opening of the inter-cellular junction pore complexes, as is shown in Wapnir et al. (1997), which would the basis for the observed permeability of cationic-cataniononeutral hydrophiles across the transiently widened inter-epithelial junction complexes of the fluid-intestinal barrier (FIB), particularly in presence of additional cationic charges (monovalent and multivalent cations) (Epler et al. 2003; Desjeux et al. 1980; Van Campen and Gross 1969).

In the case of cationic small molecule hydrophiles, the presence of singular cationic charge results in a hydrophile with a greater amount of hydrophilicity for its size than the presence of singular anionicity: Cationic hydrophiles become too hydrophilic for size to permeate across tight junction pore complexes in between a HOWPC-to-vdWD ratio of -1.0 (4-aminopyridine) and -1.8 *nm*^*-*1^ (edrophonium) at which the size for permeation is at a nadir of 0.55 nanometers, and become too hydrophilic for size to permeate across inter-epithelial junction pore complexes in between a HOWPC-to-vdWD ratio of -7.5 (methylammonium) and -8.7 *nm*^*-1*^ (glucosamine at acidic pH) at which the size for permeation is at a nadir of 0.66 nanometers. The finding of this study that cationic small molecule hydrophile, 4-aminopyridine (HOWPC-to-vdWD ratio: -1.0 *nm*^*-1*^; vdWD: 0.55 nm), is permeable across the BBB through zona occludens tight junction pore complexes can be correlated with that of *in vivo* physiologic studies in which intravenously administered 4-aminopyridine has been shown to cause altered cognition (van Diemen et al. 1992; Bever et al. 1994), whereby the side effect of altered sensorium is only attributable to the permeation of 4-aminopyridine across the BBB through the zona occludens tight junction complex pores directly into the brain parenchyma itself, whereas the permeable fraction across the BVCSFB (Pratt et al. 1995) rapidly diffuses through the CNS parenchymal interstitium via convective forces analogous to that of cations, Na+ and Ca2+. The finding of this study that cationic small molecule hydrophile, choline (HOWPC-to-vdWD ratio: -6.9 *nm*^*-1*^; vdWD: 0.61 nm), is permeable across the BVCSFB and the FIB through the inter-epithelial junction pore complexes, but not across the BBB, can also be correlated with that of *in vivo* physiologic studies in which intravenously or orally administered small molecule hydrophile [2H4]choline has been shown to incorporate into endogenous phospholipids, including those of the CNS parenchymal cells (Hanin and Schuberth 1974; Jope and Jenden 1979).

For cationic-anionic small molecule hydrophiles, the presence of cationic and anionic charges partially separated in molecular space results in a hydrophile without significant un-opposed positive or negative charge, but which is not cataniononeutral: Cationic-anionic hydrophiles become too hydrophilic for size to permeate across tight junction pore complexes in between a HOWPC-to-vdWD ratio of -3.7 (gamma-aminobutyric acid) and -6.3 *nm*^*-*1^ (tetrodotoxin) at which the size for permeation is at a nadir of 0.57 nanometers, and become too hydrophilic for size to permeate across inter-epithelial junction pore complexes in between a HOWPC-to-vdWD ratio of -7.2 (phosphocholine) and -12.0 *nm*^*-1*^ (creatine phosphate) at which the size for permeation is at a nadir of 0.67 nanometers.

The finding of this study that cationic-anionic small molecule hydrophile, gamma-aminobutyric acid (GABA) (HOWPC-to-vdWD ratio: -3.7 *nm*^*-1*^; vdWD: 0.57 nm), is permeable across the BBB through zona occludens tight junction pore complexes can be correlated with that of *in vivo* physiologic studies in which intravenously administered GABA has been shown to enter the CNS with a saturable component to its entry into the CNS with increasing plasma levels (Loscher and Frey 1982), which would not expected to be the case for a small molecule hydrophile such as GABA, as it permeates without restriction across the blood-ventricular cerebrospinal fluid barrier (BVCSFB). The finding of this study that cationic small molecule hydrophile, tetrodotoxin (HOWPC-to-vdWD ratio: -6.3 *nm*^*-1*^; vdWD: 0.77 nm), is permeable across the BVCSFB, and predictably across the FIB, through the inter-epithelial junction pore complexes, but not across the BBB, can also be correlated with that of *in vivo* physiologic studies in which intravenously administered tetrodotoxin has been shown to cause CNS hypothermia (Clark and Coldwell 1973), which is attributable to its permeation across the BVCSFB into the CSF for effect on the posteromedial hypothalamus (Rodriguez et al. 2010).

### Permeation thresholds of cations and anions across endothelial and epithelial junctional pore complexes: Monovalent cations and anions

Based on the findings of this study, there are biological thresholds for the permeation of cations and anions across endothelial and epithelial barrier junctional pore complexes in the physiologic state, which can be characterized on the basis of cationicity or anionicity per molecular size as per the Cationization-to-Atomic Diameter (CI-to-AD ratio; nm^-1^) and the Anionization-to-Atomic Diameter (AI-to-AD ratio; nm^-1^) ratios: The permeation threshold for endogenous cations across tight junction pore complexes is between a CI-to-AD ratio of +2.20 (K+) and +2.69 (Na+), and when non-endogenous CH3Hg+, a compact heavy metal cation with its CH3 close to its center of gravity, in between a CI-to-AD ratio of +2.38 (CH3Hg+) and +2.69 (Na+), whereby sodium cation (Na+) is not permeable across tight junction pore complexes, while the permeation threshold for endogenous cations across inter-epithelial junction pore complexes is between a CI-to-AD ratio of +5.08 (Ca2+) and +6.25 (Mg2+), and when non-endogenous Pb2+, a heavy metal cation is taken into consideration, in between a CI-to-AD ratio of +5.71(Pb2+) and +6.25 (Mg2+), whereby magnesium cation (Mg2+) is not permeable across inter-epithelial junction pore complexes; and in the case of anions, the permeation threshold across tight junction pore complexes is between a AI-to-AD ratio of -4.17 (Br-) and -4.90 (Cl-), whereby chloride anion (Cl-) is not permeable across tight junction pore complexes, while the permeation threshold across inter-epithelial junction pore complexes is between a AI-to-AD ratio of -4.90 (Cl-) and -7.81 (F-), whereby fluoride anion (F-), whereby fluoride anion is not permeable across inter-epithelial junction pore complexes.

The findings of this study on the permeation thresholds of cations and anions across junctional pore complexes in the physiologic state can be correlated to the findings of *in vivo* physiologic studies in which the CSF and brain parenchyma accumulation of intraperitoneally or intravenously administered cations and anions has been studied (Davson 1955; Davson and Welch 1971). In Davson (1955), the CSF and aqueous humor accumulation of cations and anions was studied, in addition to the CSF and aqueous humor accumulation of polyneutral hydrophile glucose and other small biomolecules, and the CSF and the aqueous humor concentrations of radioactive test cations, 24Na+ and 42K+, radioactive anions, 82Br- and 131I- as well as Cl- (via precipitation by Ag+) were measured at the experimental endpoint, and the CSF-to-plasma and aqueous humor-to-plasma concentration ratios determined; (Davson and Welch 1971) is a subsequent study by the senior investigator, in which the accumulation of radioactive test cations, 24Na+ and 42K+, and anion, 36Cl- in both the CSF, as well as the brain parenchyma interstitial space, was studied at various time points and the data modeled based on different CSF turnover rates. As the findings of these two *in vivo* physiologic studies provide experimental evidence in support of the findings of this study on the permeation thresholds for cations and anions across endothelial and epithelial barrier junctional pore complexes determined *in silico*, the specific findings of the two *in vivo* physiologic studies are called upon in the ensuing discussion where applicable.

Based on the findings in Figures 1 and 3 (Davson and Welch 1971), it can be appreciated that Na+ (CI-to-AD ratio: + 2.69 nm^**-1**^; Diameter: 0.372 nm) and Cl- (AI-to-AD ratio: -4.90 nm^**-1**^; Diameter: 0.204 nm) there is a delay in the accumulation of Na+ and Cl- in the CSF as compared to the accumulation of the respective cations in the CSF, which is attributable to their unrestricted permeation across the inter-epithelial junction pore complexes of the choroid plexus epithelium of the BVCSFB, in order to then accumulate in the brain parenchyma interstitium, and also implies that both Na+ and Cl- are restricted to permeation across the zona occludens tight junction pore complexes of the cerebral blood capillary microvasculature blood-brain barrier (BBB); this observation is also supported by the findings in Table one of Davson and Welch (1971) on the flow rates (cm/sec) of 24Na+, 36Cl-, 42K+ and [32S]thiourea into the brain parenchyma demonstrating that the transcapillary flow rates of Na+ and Cl- into the brain parenchyma are an order of magnitude lower than those of K+ (CI-to-AD ratio: + 2.20 nm^**-1**^; Diameter: 0.472 nm) and [32S]thiourea (HOWPC-to-vdWD ratio: -1.1 *nm*^***-1***^; vdWD: 0.48 nm), a neutral hydrophile, K+ and thiourea. This further implies that K+ and thiourea must accumulate into the brain parenchyma across both the BVCSFB and the BBB, by way of the both junctional pore complexes, inter-epithelial and occludens, while Na+ (CI-to-AD ratio: + 2.69 nm^**-1**^; Diameter: 0.372 nm) and Cl- (AI-to-AD ratio: -4.90 nm^**-1**^; Diameter: 0.204 nm) are permeable to only to the inter-epithelial junction pore complexes.

The findings of Davson (1955), in support this study’s determinations of permeation potentials of cations and anions across junctional pore complexes and are of significance to realizing the principal permeation routes for cations, 24Na+ and 42K+, and anions, Cl- (AI-to-AD ratio: -4.90 nm^**-1**^; Diameter: 0.204 nm), 82Br- (AI-to-AD ratio: -4.17 nm^**-1**^; Diameter: 0.240 nm) and 131I- (AI-to-AD ratio: -3.57 nm^**-1**^; Diameter: 0.280 nm), importantly, in context of the likelihood for intracellular permeation through transmembrane protein channel pores. Since Davson (1955), in essence, is a study of the permeation of the respective cations and anions through the milieu of the choroid plexus, which is cellular (Nataf et al. 2006), in comparison to in the aqueous humor, which is acellular (Wu et al. 1992), both of which are arterially supplied by diaphragm fenestrated blood capillaries which permit the unrestricted permeation of cations and anions, this important difference makes it possible to discern the contribution of intracellular permeation and intracellular uptake in the cellular milieu of the choroid plexus prior to inter-epithelial permeation into the CSF in comparison to into that which occurs directly into the aqueous humor. Therefore, with this knowledge, the findings of Table one (Davson 1955) can be understood as follows: (1) The reason that both Na+ (CI-to-AD ratio: + 2.69 nm^**-1**^; Diameter: 0.372 nm) and Cl- (AI-to-AD ratio: -4.90 nm^**-1**^; Diameter: 0.204 nm) achieve almost equivalent CSF-to-plasma and aqueous humor-to-plasma concentration ratios of 1.03 (Na+ CSF-to-plasma) and 1.21 (Cl- CSF-to-plasma) and 0.96 (Na+ aqueous humor-to-plasma) and 1.015 (aqueous humor-to-plasma), respectively, is due to their relative impermeability to cellular membrane protein channel pores but their unrestricted permeation across fenestrated endothelial cell pores, and in the case of the BVCSFB, unrestricted permeation across the inter-epithelial junction pore complexes; 2) The reason that K+ (CI-to-AD ratio: + 2.20 nm^**-1**^; Diameter: 0.472 nm) achieves only ~50% of its plasma concentration in the CSF with a CSF-to-plasma concentration ratio of 0.52 (K+ CSF-to-plasma) in contrast to almost 100% of its plasma concentration in the aqueous humor with an aqueous humor-to-plasma concentration ratio of 0.52 (K+ aqueous humor-to-plasma) is due to its permeability to cellular membrane protein channel pores and intracellular permeation and intracellular uptake in the cellular milieu of the choroid plexus on the way to permeation across the inter-epithelial junction pore complexes of the BVCSFB into CSF in contrast to its direct permeation into the aqueous humor; furthermore, with respect to endothelial cells, it deserves mention that, K+ is only relatively permeable across endothelial cell membrane protein channel pores (Olesen et al. 1988); and 3) The reason that Br- (AI-to-AD ratio: -4.17 nm^**-1**^; Diameter: 0.240 nm) achieves only ~70% of its plasma concentration in the CSF with a CSF-to-plasma concentration ratio of 0.715 (Br- CSF-to-plasma) in contrast to almost 100% of its plasma concentration in the aqueous humor with an aqueous humor-to-plasma concentration ratio of 0.98 (Br- aqueous humor-to-plasma) is also due to its permeability to cellular membrane protein channel pores and intracellular permeation and uptake in the cellular milieu of the choroid plexus on the way to permeation across the inter-epithelial junction pore complexes of the BVCSFB into CSF in contrast to its direct permeation into the aqueous humor; whereas, in the case of I- (AI-to-AD ratio: -3.57 nm^**-1**^; Diameter: 0.280 nm), it achieves almost none of its plasma concentration in the CSF with a CSF-to-plasma concentration ratio of 0.004 (I- CSF-to-plasma) as well as only ~30% of its plasma concentration in the aqueous humor with an aqueous humor-to-plasma concentration ratio of 0.32 (I- aqueous humor-to-plasma), which is due to its exquisite permeability to cell membrane protein channel pores and intracellular permeation and uptake, requiring fairly high concentrations of iodide salt to be administered parentrally for I- to achieve measurable concentrations in tissues (Ahmed and Van Harreveld 1969), as I- possesses the least amount of anionization for atomic size of the anions.

In context of the findings of this study that the permeation potentials of monovalent cations and anions across endothelial and epithelial barrier pore complexes can be modeled based on knowledge of the cationization per atomic size (CI-to-AD ratio; nm^**-1**^) and anionization per atomic size (AI-to-AD ratio; nm^**-1**^) ratios, respectively, it can be surmised that cell membrane protein channel pores are almost equally as restrictive to the permeation of cations carrying a greater positive charge per atomic size than K+, and anions carrying a greater negative charge per atomic size than Cl-, that is also the case for anion, F- (AI-to-AD ratio: -7.80 nm^**-1**^; Diameter: 0.128 nm), which is not permeable to any significant extent across even inter-epithelial junction pore complexes due to its significant charge per atomic molecular size, being only permeable to epithelial barriers at toxic concentrations, and in the case of the CNS, particularly toxic to the choroid plexus and its BVCSFB, which is arterially supplied by blood capillaries lined by diaphragm fenestrated endothelial cells across which F- permeates unrestrictedly to exert fluorotoxcity to the BVCSFB itself, and then to brain structures surrounding the choroid plexus such as the hippocampi (Mullenix et al. 1995).

### Permeation thresholds of cations and anions across endothelial and epithelial junctional pore complexes: divalent endogenous cations

Based on the findings of this study, the conserved biophysical parameters of charge per size, atomic size and in the case of heavy metals, density, provide insight into the permeation potentials of divalent cations across endothelial and epithelial barriers: Divalent cations are not permeable across the inter-endothelial zona occludens tight junction pore complexes; The threshold for permeation of divalent cations across inter-epithelial junctional pore complexes begins at the amount of cationicity per molecular size ratio of the calcium, Ca2+, which has a CI-to-AD ratio of + 5.08 nm^-1^ and diameter of 0.392 nm. In the case of Ca2+, its permeation across choroid plexus epithelium inter-epithelial junctions of the BVCSFB into the ventricular CSF is limited as per the saturation of its rate of accumulation when administered systemically in its free radioactive form, 45Ca2+ (Graziani et al. 1967), which is consistent with the saturable second order aspect of restricted permeation across inter-epithelial junction pore complexes; whereas, in the case of magnesium, Mg2+, which has a CI-to-AD ratio of + 6.25 *nm*^*-1*^ and diameter of 0.320 nm, is absolutely restricted to permeation across inter-epithelial junction pore complexes, and for this reason does not accumulate in the CSF to any significant extent (Sun et al. 2009). In the case of divalent cations with greater cationicity for size including Ca2+ and Mg2+, permeation across restrictive endothelial and epithelial barriers is in their anionic hydrophile chelated forms, for example, in the case of Mg2+, as Mg2+-citrate.

These findings of this study can be correlated with the role that divalent cations have in the biological system in the physiological state, in which context, the role that Ca2+ plays in the biological system is best appreciated by applying knowledge on vertebrate musculoskeletal system physiology: Ca2+ is relatively restricted to permeation across inter-epithelial junction pore complexes, but remains freely permeable across the sub-set of arterially supplied bone blood capillaries that are diaphragm fenestrated (Cooper et al. 1966), whereby it can permeate into and associate with the osteoblastic extracellular collagenous matrix, most avidly with phosphoric acid moieties, as the polyanionicity of pyrophosphoric acid is within a hydrophilicity range of -20.2 *nm*^***-1***^ (alkaline pH) and -11.2 *nm*^***-1***^ (pH 7.4), sufficient to attract multiple Ca2+ atoms for the formation of hydroxyapatite (HCa5O13P3) over the gradient from outer-to-inner, form cortical-to-cancellous and from diaphyseal-to-epiphyseal, which, importantly, is due to the absence of an initial lymphatic drainage in the diaphyseal region which permits the deposition of Ca2+ at the highest concentration, with a graded decrease over the increasing presence of initial lymphatic drainage of periosteal fibrous tissues (reciprocal relationship) (Sarin 2010).

### Permeation thresholds of exogenous cations across endothelial and epithelial junctional pore complexes and risk for toxicity

Based on the findings of this study, Pb2+ (CI-to-AD ratio: +5.71 nm^**-1**^; Diameter: 0.350 nm) has limited potential to permeate across inter-epithelial junction pore complexes, being intermediate in cationization for size between Ca2+ (CI-to-AD ratio: +5.08 nm^**-1**^; Diameter: 0.392 nm), which is semi-permeable, and Mg2+ (CI-to-AD ratio: +6.25 nm^**-1**^; Diameter: 0.320 nm), which is impermeable, which is while methyl-mercury (CH3Hg+) (CI-to-AD ratio: +2.38 nm^**-1**^; Diameter: 0.420 nm), a compact heavy metal cation with its CH3 close to its center of gravity, has the potential to permeate across inter-endothelial tight junction pores complexes, being intermediate in cationization for size between K+ (CI-to-AD ratio: + 2.20 nm^**-1**^; Diameter: 0.472 nm) and Na+ (CI-to-AD ratio: + 2.69 nm^**-1**^; Diameter: 0.372 nm). Based on these observations of this study, the mechanisms underlying heavy metal toxicity to the biological system in the physiologic state can be understood, with attention to the presence of cationization, cationization-to-atomic diameter ratios and small absolute size of density, which are the conserved biophysical properties that govern their biodistribution and biocompartmentalization.

In the case of Lead, in its divalent heavy metal cation form of Pb2+, with cationization-to-atomic diameter ratio of +5.71 nm^-1^ with diameter: of 0.350 nm has the potential to permeate across inter-epithelial junctional pore complexes of the fluid-intestinal barrier (FIB), in its ionic form, but particularly when ingested in its oxide or chelated form when its cationicity is neutralized and at significant doses (Dieter et al. 1993), whereas, when inhaled in its ionic form, Pb2+, as aerosolized paint chip nanoparticulates or smelter emissions (Landrigan et al. 1976), there is the greater potential for permeation into systemic circulation due to prolonged cationic charge-mediated toxicity locally (Bischoff et al. 1928), to epithelial cell membranes (Shafiq Ur 2013), secondarily, resulting in inter-epithelial junction pore complex disruption (Navarro-Moreno et al. 2009), which become permissive to the inter-epithelial permeation of Pb2+ ions, resulting then in the subsequent unrestricted diffusion into pulmonary blood capillaries lined by endothelial cells interconnected by the much less restrictive macula occludens loose junction pore complexes with pore widths of 4 nanometers (Sarin 2010). The greatest risk for Pb2+ toxicity is during the developmental period *in utero* (DeLorenzo 1980; Xia and Storm 2005), which is to the central nervous system (CNS) synapses, during the period of time when, in addition to the BVCSFB, the BBB is still open, as the zona occludens tight junctions of the cerebral blood capillary lining endothelial cells do not close until later in the *in utero* period (Evans et al. 1974), the *in utero* period being a time period when the developing fetus in especially vulnerable to any superimposed maternal exposures, as during this time maternal blood Pb2+ concentrations are known to be elevated secondary to its mobilization from the cancellous bone extracellular matrix (Buchet et al. 1977), where Pb2+ (CI-to-AD ratio: +5.71 nm^**-1**^; Diameter: 0.350 nm) associates less avidly with anionic pyrophosphate moieties than does Ca2+ (CI-to-AD ratio: +5.08 nm^-1^; Diameter: 0.392 nm) due to lesser cationization per atomic diameter. Furthermore, since Pb2+ can cross continuous blood capillaries with macula occludens loose inter-endothelial junctions of the cardiac and skeletal muscle blood capillaries (blood-muscle barriers); therefore, it causes peripheral neurotoxicity at the level of the neuromuscular junctions (NMJs), during adulthood as well as later development (Landrigan et al. 1976; Schwartz et al. 1988).

The primary pathway underlying the mechanism of Pb2+’s toxicity, in its Pb2+ form, is its competitive binding of the Ca2+-binding protein calmodulin (Kursula and Majava 2007) in tissues supplied by less restrictive capillary barriers, which is likely the route of its vesicular uptake via calmodulin-rich cell membranes and intra-vesicular retention, including neuronal cell boutons (DeLorenzo 1980), which in effect, eliminates the functionally of the involved surface area; if encountered in its, lipophilic tetraethyl Lead form devoid of exterior cationicity, by either inhalation or per os intake, then it incorporates more ubiquitously into sub-cellular mitochondrial lipid bilayers (i.e. inner) disrupting respiration (personal observation), and with some delay may excorporate to disrupt intra-cellular Ca2+-Calmodulin function.

Mercury is the other heavy metal small molecule that is a common toxin (Landrigan et al. 2006), which is of particular interest as it is toxic in several forms (Hg0, Hg2+ and CH3Hg+), and differences in permeation potential of its various forms across endothelial and epithelial barrier junction pore complexes determine the nature of its biodistribution and biocompartmentalization, which is predictable on the basis of conserved biophysical properties, the absence, or presence of cationization with respect to the cationization-to-atomic diameter ratios of its forms. Toxicity from Mercury can occur by: (1) inhalation exposure to its Hg0 gaseous vapor form (CI-to-AD ratio: 0 nm^-1^; Diameter: 0.302 nm), which is oxidized in the biological system to its Hg2+ form (CI-to-AD ratio: +6.62 nm^-1^; Diameter: 0.302 nm), or by (2) gastrointestinal exposure to its cationic Hg2+ form in the concomitant presence of Cystiene (HOWPC-to-vdWD ratio: -3.4 *nm*^***-1***^; vdWD: 0.57 nm) as a sulfohydral group adduct (+Hg-[S]Cystiene[COO-] or simply +Hg-Cystiene), which is overall electroneutral, and most commonly, via environmental exposure to both its CH3-Hg+ (CI-to-AD ratio: +2.38 nm^**-1**^; Diameter: 0.420 nm) and CH3Hg-Cystiene forms via consumption of big fish muscle in which it has bioacummulated in the oceanographic food chain over time via entry as Hg2+, and intestinal absorption following conversion (ie bacterial) to +Hg-Cystiene.

In the case of exposure to the gaseous Hg0 vapor (CI-to-AD ratio: 0 nm^-1^; Diameter: 0.302 nm), it permeates unrestrictedly through cell membrane protein channel pores and across junctional complexes in its un-oxidized form (Khayat and Dencker 1984; Warfvinge et al. 1994). The oxidation of Hg0 is both extra-cellular by secreted plasma catalases (Yeung et al. 1998; Tudhope 1969), and for its cell membrane-permeable un-oxidized fraction, intra-cellular by intracellular catalases (Khayat and Dencker 1984). Systemically circulating Mercury, in its divalent Hg2+ form (CI-to-AD Ratio: +6.62 nm^-1^; 0.302 nm), does not permeate across inter-epithelial junction pore complexes or inter-endothelial zona occludens tight junction pore complexes (Moller-Madsen 1990), but instead associates with the anionic glycocalyx coats of endothelial and epithelial surfaces and can interact directly with endothelial and epithelial cell membrane surface proteins, whereby it can covalently bind to electronegative sulfohydrals (S-) cysteinyl groups to form +Hg-cysteinyl-protein adducts, including those on the cysteinyl groups protruding from red blood cell membranes (Jang et al. 2011), RBC membranes readily being in constant contact with capillary wall glycocalyical matrices (Vahter et al. 1994); whereas, in the case of less restrictive endothelial barriers such as the blood-muscle barrier with macula occludens loose junctions, for example, of muscle tissue, Hg2+ is free to permeate across without restriction, and upon cysteinylation to its +Hg-Cystiene form, it readily absorbable into cells, including muscle cells. In the case of the cell membrane-permeable un-oxidized fraction of Mercury as Hg0, this fraction is intra-cellularly oxidized to Hg2+, and it binds to sulfohydral groups (S-), particularly in tissue cells rich in cytosolic cysteinyl group-containing metallothionein proteins (Shimada et al. 2005). It is worth mentioning that cationic cadmium, Cd2+, while not as heavy as Hg2+, being identical in CI-to-AD ratio to Hg2+ as well as in atomic diameter (Gruff and Koch 1990), is likely to be similar to Hg2+ in toxic potential in the physiologic state.

Furthermore, in the case of CH3Hg+ (CI-to-AD ratio: +2.38 nm^-1^; Diameter : 0.420 nm), this form of Mercury is permeable across inter-epithelial junction pore complexes, and via restriction diffusion across the inter-endothelial tight junction pore complexes, due to significantly less cationicity for size than Hg 2+. The fact that CH3Hg+ is permeable without restriction across the fluid-intestinal-barrier (FIB) inter-epithelial junction pore complexes, is important as CH3Hg+, the predominant form in fish muscle (Lemes et al. 2011), can biodistribute extensively via systemic circulation (Moller-Madsen 1990, 1991; Gyrd-Hansen 1981), particularly can diffuse into the central nervous system (CNS) parenchyma across both the BBB and BVSCFB, and then diffuse across cell membrane protein channel pores to localize intracellularly, as CH3Hg+ is only slightly more cationic for molecular size than K+ (CI-to-AD Ratio: +2.20 nm^**-1**^; Diameter: 0.472 nm), which is why Mercury in the form of CH3-Hg+ is neurotoxic, including to the BBB endothelial cells themselves (Bertossi et al. 2004). In support of these conclusions, at the *in vivo* morphologic studies of Moller-Madsen (1990, 1991) in which it is shown that CH3Hg+ deposits extensively throughout the CNS of rodents after administration via intraperitoneal and oral routes. It can be postulated that, upon permeation of barriers, the final common pathway underlying the mechanism of intracellular CH3Hg+ toxicity is genotoxicity (Grotto et al. 2009) due to its covalent binding of DNA (Maki and Ott 1981) and disruption of transcription pathways, leading to cellular instability and cell death. Exposure to significant doses of CH3Hg+ during pregnancy have resulted in offspring with significant neurodevelopmental abnormalities as was the case at Minamata Bay (Eto et al. 2010).

## Conclusions

When modeling biomolecular permeability *in silico* for the physiologic state of the biological system, certain assertions are made, and need to be accounted for, which deserve mention. The first of these assertions relate to the other contributors to trans-capillary wall permeation other than diffusion or restricted diffusion across endothelial and epithelial junction pore complexes, and particularly, to the possible contribution of the much documented phenomenon of endocytosis coupled trans-endocytosis towards the trans-capillary wall transport of small molecule hydrophiles, and in the case of macromolecules, for permeation across continuous and fenestrated capillary walls. The important point is that these phenomena have only been observed with cationic macromolecular tracers (De Bruyn et al. 1975, 1983; Bankston and Milici 1983; Dvorak et al. 1996), and are entirely reactive phenomena in response to macromolecular cationicity to the endothelium (and epithelium), which have been observed, particularly in the case of fenestrated capillary endothelia with a thin anionic glycocalyx endo-capillary layer (Sarin 2010); therefore, in the physiologic state, endocytosis coupled trans-endocytosis (or epithelocytosis coupled trans-epithelocytosis) is not a contributor to the trans-capillary wall transport of macromolecules, and in the case of small molecule cationic hydrophiles, being only of significance to neuronal bouton-mediated neurotransmitter uptake. The second of the assertions relates to the transport of small molecule hydrophiles through endothelial and epithelial cell protoplasma via cell membrane aqueous protein channels, which also does not occur in the physiologic state, being only of significance to diffusional exchange of small neutral hydrophiles (ie urea, water and gases) in between the extracellular and intracellular compartments.

Physiologic measurements on diffusional permeation of small biomolecules across capillary walls to-date have provided valuable insight into relative permeabilities of small hydrophilic solute molecules across various endothelial barriers, based on which it can be reasoned that the aqueous pores in between endothelial cells and through endothelial cells are the routes for the trans-capillary wall permeation of small molecule hydrophiles; however, these studies have not considered that the biophysical properties of small hydrophiles may be the important determinants of permeation potential across barriers, for which reason it has been difficult to identify the exact permeation thresholds for small molecule hydrophiles for diffusion across restrictive barriers such as the blood-brain barrier (BBB) with endothelium inter-connected by zona occludens tight junctions, or for epithelial barriers such as the blood-ventricular cerebrospinal fluid barrier (BVCSFB) and the fluid-intestinal barrier (FIB) (Additional file 12: Table S12. Permeation Thresholds for Hydrophile Small Biomolecules across Zona Occludens Tight Junction Pore Complexes; Additional file 13: Table S13. Permeation Thresholds for Hydrophile Small Biomolecules across Inter-epithelial Junction Pore Complexes; Additional file 14: Table S14. Permeation Thresholds of Pore Sizes for Hydrophilic Biomolecules across Blood Capillary Wall Endothelial Fenstrations and Pore Complexes versus Inter-epithelial Pore Complexes in Nanometers).

In this study, the permeation thresholds of small molecule hydrophiles across microvascular capillary wall across inter-endothelial junction complexes of the zona occludens tight junction type and across inter-epithelial junction complexes have been modeled *in silico* on the basis of conserved biomolecular properties: (1) Categorizing small molecule hydrophiles by the character of hydrophilicity distribution over molecular space, as anionic, anionic-cataniononeutral, pure polyneutral, neutral-cataniononeutral/cataniononeutral, mixed polyneutral, neutral, cationic, cationic-cataniononeutral and cationic-anionic, and modeling their permeation potentials based on predicted overall hydrophilicity for molecular size as per their predicted hydrophilic octanol-to-water partition coefficient (HOWPC)–to-van der Waals diameter (vdWD) ratios (*nm*^*-1*^), and (2) 2-D plotting of their predicted hydrophilic octanol-to-water partition coefficient (HOWPC; unitless) on the y-axis in context of van der Waals diameter (vdWD; nm) on the x-axis, which serves out to separate the relative contributions of predicated overall hydrophilicity and molecular size towards inter-endothelial or inter-epithelial permeability.

In the case of biologically-relevant metal and non-metal cations and anions, it has been determined that cations and anions more or less are of equivalent hydrophilicities, and that the primary determinant of biological interaction are the amount of charge per molecular size in context of the molecular size and weight density, rather than the amount of absolute hydrophilicity per molecular size.. Based on this knowledge, the permeation thresholds of metal and non-metal cations and anions across endothelial and epithelial barriers have been modeled *in silico viz a viz* ionization-to-atomic diameter ratios as these ratios accurately represent the distribution of charge homogenously over the element’s volume for cations (Cationization-to-Atomic Diameter ratio: CI-to-AD ratio; nm^-1^) and for anions (Anionization-to-Atomic Diameter ratio: AI-to-AD ratio; nm^-1^), respectively, based on which important insight has been gained into the permeation potentials of anions, cations and cationic heavy metal forms across endothelial and epithelial barriers.

This knowledge is widely applicable, including towards the development of systematic approaches to small molecule therapeutic design by predicting *a priori* exactly what the limitations to epithelial and endothelial barrier permeation will be in context of the potential for biodistribution toxicity.

## Additional files

Additional file 1: Table S1. Panel A. Hydrophiles: Anionic through Tight Junction Pore Complexes; Panel B. Hydrophiles: Anionic through Inter-Epithelial Pore Complexes. Additional file 2: Table S2. Panel A. Hydrophiles: Anionic-Cationoneutral through Tight Junction Pore Complexes; Panel B. Hydrophiles: Anionic-Cationoneutral through Inter-Epithelial Pore Complexes. Additional file 3: Table S3. Panel A. Hydrophiles: Pure Polyneutral through Tight Junction Pore Complexes; Panel B. Hydrophiles: Pure Polyneutral through Inter-Epithelial Pore Complexes. Additional file 4: Table S4. Panel A. Hydrophiles: Neutral-Cationoneutral through Tight Junction Pore Complexes; Panel B. Hydrophiles: Neutral-Cationoneutral through Inter-Epithelial Pore Complexes. Additional file 5: Table S5. Panel A. Hydrophiles: Mixed Polyneutral through Tight Junction Pore Complexes; Panel B. Mixed Hydrophiles: Mixed Polyneutral through Inter-Epithelial Pore Complexes. Additional file 6: Table S6. Panel A. Hydrophiles: Neutral through Tight Junction Pore Complexes; Panel B. Hydrophiles: Neutral through Inter-Epithelial Pore Complexes. Additional file 7: Table S7. Panel A. Hydrophiles: Cationic-Cationoneutral through Tight Junction Pore Complexes; Panel B. Hydrophiles: Cationic- Cationoneutral through Inter-Epithelial Pore Complexes.) Additional file 8: Table S8. Panel A. Hydrophiles: Cationic through Tight Junction Pore Complexes; Panel B. Hydrophiles: Cationic through Inter-Epithelial Pore Complexes. Additional file 9: Table S9. Panel A. Hydrophiles: Cationic-Anionic through Tight Junction Pore Complexes; Panel B. Hydrophiles: Cationic-Anionic through Inter-Epithelial Pore Complexes. Additional file 10: Table S10. Panel A. Endogenous and Non-endogenous Non-Metals and Metals through Zona Occludens Tight Junction Pore Complexes; Panel B. Endogenous and Non-endogenous Non-Metals and Metals through Inter-Epithelial Pore Complexes. Additional file 11: Table S11. Panel A. Biological Halogens through Zona Occludens Tight Junction Pore Complexes; Panel B. Biological Halogens through Inter-Epithelial Pore Complexes. Additional file 12: Table S12. Permeation Thresholds for Hydrophile Small Biomolecules across Zona Occludens Tight Junction Pore Complexes. Additional file 13: Table S13. Permeation Thresholds for Hydrophile Small Biomolecules across Inter-Epithelial Pore Complexes. Additional file 14: Table S14. Permeation Thresholds of Pore Sizes for Hydrophilic Biomolecules across Blood Capillary Wall Endothelia in Nanometers.

## Abbreviations

poHOWPC or HOWPC (*nm*^*−1*^): Predicted overall hydrophilic octanol-to-water partition coefficient
vdWD (nm): van der Waals Diameter
HOWPC-to-vdWD ratio (*nm*^*−1*^): Predicted hydrophilic octanol-to-water partition coefficient-to-molecular diameter ratio
vdWD @ MAXimum HOWPC-to-vdWD (nm): vdWD for permeable hydrophile at maximum predicted hydrophilic octanol-to-water partition coefficient-to-molecular diameter ratio
vdWD @ MINimum HOWPC-to-vdWD (nm): vdWD for permeable hydrophile at minimum predicted hydrophilic octanol-to-water partition coefficient-to-molecular diameter ratio
CI-to-AD ratio (nm^−1^): Predicted cationization-to-atomic diameter ratio
AI-to-AD ratio (nm^−1^): Predicted anionization-to-atomic diameter ratio
