# Supplementary material for: Permeation thresholds for hydrophilic small biomolecules across microvascular and epithelial barriers are predictable on basis of conserved biophysical properties
Source: In Silico Pharmacol. 2015 May 3;3:5. doi: 10.1186/s40203-015-0009-y (PMC4471070; doi:10.1186/s40203-015-0009-y)
Supplement: Additional file 1: Table S1. — Panel A. Hydrophiles: Anionic through Tight Junction Pore Complexes; Panel B. Hydrophiles: Anionic through Inter-Epithelial Pore Complexes. [file 40203_2015_9_MOESM1_ESM.pdf]

TABLE 1A. Hydrophiles: Anionic through Tight Junction Pore Complexes

	Formula	Log Pow	Pow	Log Dow	Dow	Weight (Daltons)	Volume (Ang3)	vdWD (nm)	Psa	Ionicity	Charge Distribution	Groups	HOPWC-to-vdWD Ratio (per nm [nm-1])
Pyrophosphoric Acid @ Alkaline pH	O7P2	-1.44	3.631E-02	-11.50	3.162E-12	174	100	0.57	136	Anionic	IS 2- 2-	PO4 2-, PO4 2-	-20.2
Foscarnet Chelate	CH3O5P	-0.83	1.479E-01	-9.00	1.000E-09	125	84	0.54	94	Anionic	3-	PO-, COO-	-16.8
Citrate (Citric Acid) Chelate	C6H8O7	-1.32	4.786E-02	-11.00	1.000E-11	192	154	0.66	132	Anionic	3-	COO- X3	-16.7
Phosphate (Phosphoric Acid)	HPO4 2-	-1.02	9.550E-02	-5.95	1.122E-06	95	57	0.47	86	Anionic	2-	PO4 2-	-12.6
Sulfate	O4S 2-	-0.84	1.445E-01	-5.54	2.884E-06	96	60	0.48	80	Anionic	2-	SO4 2-	-11.5
Pyrophosphoric Acid @ pH 7.4	H2O7P2	-1.44	3.631E-02	-6.35	4.467E-07	176	100	0.57	136	Anionic	IS 1- 1-	PO4 1-, PO4 1-	-11.2
Sialic Acid	C11H19NO9	-3.95	1.122E-04	-7.40	3.981E-08	309	268	0.79	185	Anionic	1-	OH X5, COO - X1	-9.4
Thiophosphate (Thiophosphoric Acid)	H3O3PS	-0.13	7.413E-01	-4.80	1.585E-05	114	74	0.51	61	Anionic	2-	S, PO 1- X2	-9.3
Glucuronic Acid	C6H10O7	-2.60	2.512E-03	-5.60	2.512E-06	194	157	0.66	127	Anionic	1-	OH X4, COO- X1	-8.5
Carbamic Acid	CH3NO2	-0.56	2.754E-01	-3.80	1.585E-04	61	50	0.45	63	Anoinic	1-	COO-	-8.4
Cyanate	CNO	-0.54	2.884E-01	-3.30	5.012E-04	42	34	0.40	47	Anionic	1-	O-	-8.3
Cyclic Ascorbate Ester	C6H7O6	-1.91	1.230E-02	-5.20	6.310E-06	176	140	0.64	107	Anionic	1-	OH X3, O-	-8.2
Acetate (Acetic Acid)	C2H3O2	-0.22	6.026E-01	-3.55	2.818E-04	59	54	0.46	40	Anionic	1-	COO-	-7.7
Lactate (Lactic Acid)	C3H5O3	-0.47	3.388E-01	-3.70	1.995E-04	89	79	0.53	60	Anionic	1-	OH, COO-	-7.0
Hydrogen Sulfate	HO4S 1-	-0.8	1.445E-01	-3.20	6.310E-04	96	60	0.48	80	Anionic	1-	HSO4-	-6.7
Methyl(Flouro)phosphonic acid	CH4FO2P	-0.54	2.884E-01	-2.85	1.413E-03	98	69	0.50	37	Anionic	1-	PO-	-5.7
P-aminohippuric acid	C9H10N2O3	-0.80	1.585E-01	-3.60	2.512E-04	194	168	0.68	92	Anionic	1-	COO-	-5.3
Perchlorate (Perchloric Acid)	ClO4	-2.32	4.786E-03	-2.32	4.786E-03	99	57	0.47	74	Anionic	1-	O-	-4.9
Mg-Citrate	C6H8O7	-1.32	4.786E-02	n/a	n/a	216	135	0.63	--	Anionic	3-(2+) [=1-]	COO- X3 [Mg2+]	-2.1
Mg-Foscarnet	CH3O5P	-0.83	1.479E-01	n/a	n/a	149	74	0.51	--	Anionic	3-(2+) [=1-]	PO-X2, COO- [Mg2+]	-1.6
Probenacid	C13H19NO4S	2.44	2.754E+02	-0.85	1.413E-01	285	261	0.78	75	Anionic	1-	COO-	-1.1

Red = Not Permeable
Green = Permeable

TABLE 1B. Hydrophiles: Anionic through Inter-Epithelial Pore Complexes

	Formula	Log Pow	Pow	Log Dow	Dow	Weight (Daltons)	Volume (Ang3)	vdWD (nm)	Psa	Ionicity	Charge Distribution	Groups	HOPWC-to-vdWD Ratio (per nm [nm-1])
Pyrophosphoric Acid @ Alkaline pH	O7P2	-1.44	3.631E-02	-11.50	3.162E-12	174	100	0.57	136	Anionic	IS 2- 2-	PO4 2-, PO4 2-	-20.2
Foscarnet Chelate	CH3O5P	-0.83	1.479E-01	-9.00	1.000E-09	125	84	0.54	94	Anionic	3-	PO-, COO-	-16.8
Citrate (Citric Acid) Chelate	C6H8O7	-1.32	4.786E-02	-11.00	1.000E-11	192	154	0.66	132	Anionic	3-	COO- X3	-16.7
Phosphate (Phosphoric Acid)	HPO4 2-	-1.02	9.550E-02	-5.95	1.122E-06	95	57	0.47	86	Anionic	2-	PO4 2-	-12.6
Sulfate	O4S 2-	-0.84	1.445E-01	-5.54	2.884E-06	96	60	0.48	80	Anionic	2-	SO4 2-	-11.5
Pyrophosphoric Acid @ pH 7.4	H2O7P2	-1.44	3.631E-02	-6.35	4.467E-07	176	100	0.57	136	Anionic	IS 1- 1-	PO4 1-, PO4 1-	-11.2
Sialic Acid	C11H19NO9	-3.95	1.122E-04	-7.40	3.981E-08	309	268	0.79	185	Anionic	1-	OH X5, COO - X1	-9.4
Thiophosphate (Thiophosphoric Acid)	H3O3PS	-0.13	7.413E-01	-4.80	1.585E-05	114	74	0.51	61	Anionic	2-	S, PO 1- X2	-9.3
Glucuronic Acid	C6H10O7	-2.60	2.512E-03	-5.60	2.512E-06	194	157	0.66	127	Anionic	1-	OH X4, COO- X1	-8.5
Carbamic Acid	CH3NO2	-0.56	2.754E-01	-3.80	1.585E-04	61	50	0.45	63	Anoinic	1-	COO-	-8.4
Cyanate	CNO	-0.54	2.884E-01	-3.30	5.012E-04	42	34	0.40	47	Anionic	1-	O-	-8.3
Cyclic Ascorbate Ester	C6H7O6	-1.91	1.230E-02	-5.20	6.310E-06	176	140	0.64	107	Anionic	1-	OH X3, O-	-8.2
Acetate (Acetic Acid)	C2H3O2	-0.22	6.026E-01	-3.55	2.818E-04	59	54	0.46	40	Anionic	1-	COO-	-7.7
Lactate (Lactic Acid)	C3H5O3	-0.47	3.388E-01	-3.70	1.995E-04	89	79	0.53	60	Anionic	1-	OH, COO-	-7.0
Hydrogen Sulfate	HO4S 1-	-0.8	1.445E-01	-3.20	6.310E-04	96	60	0.48	80	Anionic	1-	HSO4-	-6.7
Methyl(Flouro)phosphonic acid	CH4FO2P	-0.54	2.884E-01	-2.85	1.413E-03	98	69	0.50	37	Anionic	1-	PO-	-5.7
P-aminohippuric acid	C9H10N2O3	-0.80	1.585E-01	-3.60	2.512E-04	194	168	0.68	92	Anionic	1-	COO-	-5.3
Perchlorate (Perchloric Acid)	ClO4	-2.32	4.786E-03	-2.32	4.786E-03	99	57	0.47	74	Anionic	1-	O-	-4.9
Mg-Citrate	C6H8O7	-1.32	4.786E-02	n/a	n/a	216	135	0.63	--	Anionic	3-(2+) [=1-]	COO- X3 [Mg2+]	-2.1
Mg-Foscarnet	CH3O5P	-0.83	1.479E-01	n/a	n/a	149	74	0.51	--	Anionic	3-(2+) [=1-]	PO-X2, COO- [Mg2+]	-1.6
Probenacid	C13H19NO4S	2.44	2.754E+02	-0.85	1.413E-01	285	261	0.78	75	Anionic	1-	COO-	-1.1

Red = Not Permeable
Green = Permeable
