# Supplementary material for: Permeation thresholds for hydrophilic small biomolecules across microvascular and epithelial barriers are predictable on basis of conserved biophysical properties
Source: In Silico Pharmacol. 2015 May 3;3:5. doi: 10.1186/s40203-015-0009-y (PMC4471070; doi:10.1186/s40203-015-0009-y)
Supplement: Additional file 2: Table S2. — Panel A. Hydrophiles: Anionic-Cationoneutral through Tight Junction Pore Complexes; Panel B. Hydrophiles: Anionic-Cationoneutral through Inter-Epithelial Pore Complexes. [file 40203_2015_9_MOESM2_ESM.pdf]

**TABLE 2A. Hydrophiles: Anionic-Catanioneutral through Tight Junction Pore Complexes**

| | Formula | Log Pow | Pow | Log Dow | Dow | Weight (Daltons) | Volume (Ang3) | vdWD (nm) | Psa | Ionicity | Charge Distribution | Groups | HOWPC-to-vdWD Ratio (per nm [nm-1]) |
|---|---|---|---|---|---|---|---|---|---|---|---|---|---|
| Phosphoserine | C3H8NO6P | -3.18 | 6.61E-04 | -8.00 | 1.000E-08 | 185 | 138 | 0.63 | 130 | Polyanionic-Catanioneutral | S 1- (1-) IS 1+, 1- | PO4 2-, NH3+, COO- | -12.6 |
| 2-(2,3-dicarboxycyclopropyl)glycine (DCPG, DCG) | C7H9NO6 | -4.80 | 1.58E-05 | -8.27 | 5.370E-09 | 203 | 163 | 0.67 | 138 | Anionic-Catanioneutral | S 2- IS 1+, 1- | COO- X2, NH3+, COO- | -12.4 |
| Aspartate (Aspartic Acid) | C5H8NO4 | -2.65 | 7.94E+04 | -6.00 | 1.000E-06 | 133 | 112 | 0.59 | 100 | Anionic-Catanioneutral | S 1- IS 1+, 1- | COO-, NH3+, COO- | -10.2 |
| Glutathione | C10H17N3O6S | -4.90 | 7.94E+04 | -7.40 | 3.981E-08 | 307 | 261 | 0.78 | 159 | Anionic-Catanioneutral | S 1- IS 1+, 1- | COO-, [SH], NH3+, COO- | -9.5 |
| Glutamate (Glutamic Acid) | C5H8NO4 | -2.40 | 7.94E+04 | -5.50 | 3.162E-06 | 146 | 127 | 0.62 | 63 | Anionic-Catanioneutral | S 1- IS 1+, 1- | COO-[CH2], NH3+, COO- | -8.9 |
| N-Methyl-D-Aspartate (NMDA Acid) | C5H9NO4 | -3.27 | 5.370E-04 | -5.40 | 3.981E-06 | 147 | 130 | 0.62 | 87 | Anionic-Catanioneutral | S 1- PS 1+, 1- | COO-, CH3-NH2(+)-CH2, COO- | -8.7 |
| Kainate (Kainic acid) | C10H15NO4 | -2.36 | 4.365E-03 | -5.00 | 1.000E-05 | 213 | 196 | 0.71 | 86 | Anionic-Catanioneutral | S 1- PS 1+, 1- | COO-, -CH2-NH2(+)-CH2-, COO- | -7.0 |

Red = Not Permeable
Green = Permeable

**TABLE 2B. Hydrophiles: Anionic-Catanioneutral through Inter-Epithelial Pore Complexes**

| | Formula | Log Pow | Pow | Log Dow | Dow | Weight (Daltons) | Volume (Ang3) | vdWD (nm) | Psa | Ionicity | Charge Distribution | Groups | HOWPC-to-vdWD Ratio (per nm [nm-1]) |
|---|---|---|---|---|---|---|---|---|---|---|---|---|---|
| Phosphoserine | C3H8NO6P | -3.18 | 6.61E-04 | -8.00 | 1.000E-08 | 185 | 138 | 0.63 | 130 | Polyanionic-Catanioneutral | S 1- (1-) IS 1+, 1- | PO4 2-, NH3+, COO- | -12.6 |
| 2-(2,3-dicarboxycyclopropyl)glycine (DCPG, DCG) | C7H9NO6 | -4.80 | 1.58E-05 | -8.27 | 5.370E-09 | 203 | 163 | 0.67 | 138 | Anionic-Catanioneutral | S 2- IS 1+, 1- | COO- X2, NH3+, COO- | -12.4 |
| Aspartate (Aspartic Acid) | C5H8NO4 | -2.65 | 7.94E+04 | -6.00 | 1.000E-06 | 133 | 112 | 0.59 | 100 | Anionic-Catanioneutral | S 1- IS 1+, 1- | COO-, NH3+, COO- | -10.2 |
| Glutathione | C10H17N3O6S | -4.90 | 7.94E+04 | -7.40 | 3.981E-08 | 307 | 261 | 0.78 | 159 | Anionic-Catanioneutral | S 1- IS 1+, 1- | COO-, [SH], NH3+, COO- | -9.5 |
| Glutamate (Glutamic Acid) | C5H8NO4 | -2.40 | 7.94E+04 | -5.50 | 3.162E-06 | 146 | 127 | 0.62 | 63 | Anionic-Catanioneutral | S 1- IS 1+, 1- | COO-[CH2], NH3+, COO- | -8.9 |
| N-Methyl-D-Aspartate (NMDA Acid) | C5H9NO4 | -3.27 | 5.370E-04 | -5.40 | 3.981E-06 | 147 | 130 | 0.62 | 87 | Anionic-Catanioneutral | S 1- PS 1+, 1- | COO-, CH3-NH2(+)-CH2, COO- | -8.7 |
| Kainate (Kainic acid) | C10H15NO4 | -2.36 | 4.365E-03 | -5.00 | 1.000E-05 | 213 | 196 | 0.71 | 86 | Anionic-Catanioneutral | S 1- PS 1+, 1- | COO-, -CH2-NH2(+)-CH2-, COO- | -7.0 |

Red = Not Permeable
Green = Permeable
