# Supplementary material for: Permeation thresholds for hydrophilic small biomolecules across microvascular and epithelial barriers are predictable on basis of conserved biophysical properties
Source: In Silico Pharmacol. 2015 May 3;3:5. doi: 10.1186/s40203-015-0009-y (PMC4471070; doi:10.1186/s40203-015-0009-y)
Supplement: Additional file 3: Table S3. — Panel A. Hydrophiles: Pure Polyneutral through Tight Junction Pore Complexes; Panel B. Hydrophiles: Pure Polyneutral through Inter-Epithelial Pore Complexes. [file 40203_2015_9_MOESM3_ESM.pdf]

TABLE 3A. Hydrophiles: Pure Polyneutral Sugars through Tight Junction Pore Complexes

	Formula	Log Pow	Pow	Log Dow	Dow	Weight (Daltons)	Volume (Ang3)	vdWD (nm)	Psa	Ionicity	Charge Distribution	Groups	HOWPC-to-vdWD Ratio (per nm [nm-1])
Raffinose Trisaccharide Sugar	C18H32O16	-6.30	5.012E-07	n/a	n/a	504	424	0.92	270	Polyneutral	n/a	OH X11	-6.8
Lactitol Disacchride Sugar	C12H24O11	-5.50	3.162E-06	n/a	n/a	344	300	0.82	200	Polyneutral	n/a	OH X8	-6.7
Sucrose Disaccharide Sugar	C12H22O11	-4.53	2.951E-05	n/a	n/a	342	289	0.81	190	Polyneutral	n/a	OH X8	-5.6
Mannitol Monosaccharide Sugar	C6H14O6	-3.73	1.862E-04	n/a	n/a	182	165	0.67	121	Polyneutral	n/a	OH X6	-5.5
cGlucose Sugar	C6O5H10	-2.93	1.175E-03	n/a	n/a	180	154	0.66	110	Polyneutral	n/a	OH X5	-4.5
meso-Erythritol Sugar	C4H10O4	-2.47	3.388E-03	n/a	n/a	122	114	0.59	81	Polyneutral	n/a	OH X4	-4.2
Ribose Sugar	C3H8O4	-2.20	6.310E-03	n/a	n/a	150	128	0.62	90	Polyneutral	n/a	OH X4	-3.6
Glycerol Sugar	C3H8O3	-1.84	1.445E-02	n/a	n/a	92	88	0.55	140	Polyneutral	n/a	OH X3	-3.4
Ethylene Glycol Sugar	C2H6O2	-1.21	6.166E-02	n/a	n/a	62	62	0.48	40	Polyneutral	n/a	OH X2	-2.5

Red = Not Permeable
Green = Permeable

TABLE 3B. Hydrophiles: Pure Polyneutral Sugars through Inter-Epithelial Pore Complexes

	Formula	Log Pow	Pow	Log Dow	Dow	Weight (Daltons)	Volume (Ang3)	vdWD (nm)	Psa	Ionicity	Charge Distribution	Groups	HOWPC-to-vdWD Ratio (per nm [nm-1])
Raffinose Trisaccharide Sugar	C18H32O16	-6.30	5.012E-07	n/a	n/a	504	424	0.92	270	Polyneutral	n/a	OH X11	-6.8
Lactitol Disacchride Sugar	C12H24O11	-5.50	3.162E-06	n/a	n/a	344	300	0.82	200	Polyneutral	n/a	OH X8	-6.7
Sucrose Disaccharide Sugar	C12H22O11	-4.53	2.951E-05	n/a	n/a	342	289	0.81	190	Polyneutral	n/a	OH X8	-5.6
Mannitol Monosaccharide Sugar	C6H14O6	-3.73	1.862E-04	n/a	n/a	182	165	0.67	121	Polyneutral	n/a	OH X6	-5.5
cGlucose Sugar	C6O5H10	-2.93	1.175E-03	n/a	n/a	180	154	0.66	110	Polyneutral	n/a	OH X5	-4.5
meso-Erythritol Sugar	C4H10O4	-2.47	3.388E-03	n/a	n/a	122	114	0.59	81	Polyneutral	n/a	OH X4	-4.2
Ribose Sugar	C3H8O4	-2.20	6.310E-03	n/a	n/a	150	128	0.62	90	Polyneutral	n/a	OH X4	-3.6
Glycerol Sugar	C3H8O3	-1.84	1.445E-02	n/a	n/a	92	88	0.55	140	Polyneutral	n/a	OH X3	-3.4
Ethylene Glycol	C2H6O2	-1.21	6.166E-02	n/a	n/a	62	62	0.48	40	Polyneutral	n/a	OH X2	-2.5

Red = Not Permeable
Green = Permeable
