# Supplementary material for: Permeation thresholds for hydrophilic small biomolecules across microvascular and epithelial barriers are predictable on basis of conserved biophysical properties
Source: In Silico Pharmacol. 2015 May 3;3:5. doi: 10.1186/s40203-015-0009-y (PMC4471070; doi:10.1186/s40203-015-0009-y)
Supplement: Additional file 4: Table S4. — Panel A. Hydrophiles: Neutral-Cationoneutral through Tight Junction Pore Complexes; Panel B. Hydrophiles: Neutral-Cationoneutral through Inter-Epithelial Pore Complexes. [file 40203_2015_9_MOESM4_ESM.pdf]

TABLE 4A. Hydrophiles: Neutral-Catanionoeutral and Catanionoeutral through Tight Junction Pore Complexes

	Formula	Log Pow	Pow	Log Dow	Dow	Weight (Daltons)	Volume (Ang3)	vdWD (nm)	Psa	Ionicity	Charge Distribution	Groups	HOWPC-to-vdWD Ratio (per nm [nm-1])
Asparagine	C4H7NO4	-4.50	3.16E-05	**-3.60**	**2.51E-04**	132	115	**0.60**	106	Neutral-Catanionoeutral	0 IS 1+, 1-	NH3 , NH3+, COO-	-6.0
Selenocysteineic Acid	C3H7NO2Se	n/a	n/a	**-3.25**	**5.62E-04**	168	108	**0.58**	106	Neutral-Catanionoeutral	0 IS 1+, 1-	HSe, NH3+, COO-	-5.6
Serine	C3H7NO3	n/a	n/a	**-3.10**	**7.94E-04**	105	94	**0.56**	84	Neutral-Catanionoeutral	0 IS 1+, 1-	OH, NH3+, COO-	-5.5
Glutamine	C5H10N2O3	n/a	n/a	**-3.30**	**5.01E-04**	146	132	**0.62**	63	Neutral-Catanionoeutral	0 IS 1+, 1-	NH2, NH3+, COO-	-5.3
Glycine	C2H5NO2	n/a	n/a	**-2.65**	**2.24E-03**	75	68	**0.50**	63	Catanionoeutral	IS 1+, 1-	NH3+, COO-	-5.3
Citruline	C6H13N3O3	n/a	n/a	**-3.20**	**6.31E-04**	175	161	**0.67**	118	Neutral-Catanionoeutral	0 IS 1+, 1-	NH2, NH3+, COO-	-4.8
Histidine @ pH 7.4	C6H9N3O2	n/a	n/a	**-2.90**	**1.26E-03**	155	135	**0.63**	92	Neutral-Catanionoeutral	0 IS 1+, 1-	NH2, NH3+, COO-	-4.6
Threonine	C4H9NO3	n/a	n/a	**-2.70**	**2.00E-03**	119	111	**0.59**	84	Neutral-Catanionoeutral	0 IS 1+, 1-	OH, NH3+, COO-	-4.6
Alanine	C3H7NO2	n/a	n/a	**-2.10**	**7.94E-03**	89	84	**0.54**	63	Neutral-Catanionoeutral	0 IS 1+, 1-	CH3, NH3+, COO-	-3.9
Taurine	C2H7NO3S	n/a	n/a	**-2.10**	**7.94E-03**	125	102	**0.57**	80	Catanionoeutral	IS 1+, 1-	NH3+, CH2-COO-	-3.7
Cysteine	C3H7NO2S	n/a	n/a	**-1.95**	**1.12E-02**	121	103	**0.57**	63	Neutral-Catanionoeutral	0 IS 1+, 1-	SH, NH3+, COO-	-3.4
Proline	C5H9NO2	n/a	n/a	**-1.75**	**1.78E-02**	115	108	**0.58**	49	Neutral-Catanionoeutral	0 IS 1+, 1-	NH2+ (Cyclopentane), COO-	-3.0
Methionine	C5H11NO2S	n/a	n/a	**-1.40**	**3.98E-02**	149	138	**0.63**	63	Neutral-Catanionoeutral	0 IS 1+, 1-	CH2-S-CH3, NH3+, COO-	-2.2
Valine	C5H11NO2	n/a	n/a	**-1.10**	**7.94E-02**	117	119	**0.60**	63	Neutral-Catanionoeutral	0 IS 1+, 1-	CH3 X2, NH3+, COO-	-1.8
ACPC	C6H11NO2	n/a	n/a	**-0.90**	**1.26E-01**	129	125	**0.61**	64	Neutral-Catanionoeutral	1 IS 1+, 1-	COO-, NH3+, Cyclopentane	-1.5
Levodopa (L-DOPA)	C9H11NO4	n/a	n/a	**-0.90**	**1.26E-01**	197	172	**0.68**	104	Neutral-Catanionoeutral	0 IS 1+, 1-	COO-, NH3+, Hydroxylated phenol	-1.3
Leucine	C6H13NO2	n/a	n/a	**-0.70**	**2.00E-01**	131	138	**0.63**	63	Neutral-Catanionoeutral	0 IS 1+, 1-	Isopropyl-CH3, NH3+, COO-	-1.1
Tyrosine	C9H11NO3	n/a	n/a	**-0.70**	**2.00E-01**	181	164	**0.67**	83	Neutral-Catanionoeutral	0 IS 1+, 1-	Benzyl-CH2-, OH X1, NH3+, COO-	-1.0
Isoleucine	C6H13NO2	n/a	n/a	**-0.60**	**2.51E-01**	131	136	**0.63**	63	Neutral-Catanionoeutral	0 IS 1+, 1-	Propyl-CH3, NH3+, COO-	-1.0
Pregabalin	C8H17NO2	n/a	n/a	**-0.55**	**2.82E-01**	159	170	**0.68**	63	Neutral-Catanionoeutral	0 IS 1+, 1-	COO-, NH3+, Isopropyl	-0.8
ACHC	C7H13NO2	n/a	n/a	**-0.50**	**3.16E-01**	143	142	**0.64**	63	Neutral-Catanionoeutral	0 IS 1+, 1-	COO-, NH3+, Cyclohexane	-0.8
Gabapentin	C9H17NO2	n/a	n/a	**-0.50**	**3.16E-01**	171	176	**0.69**	63	Neutral-Catanionoeutral	0 IS 1+, 1-	COO-, NH3+, Cyclohexane	-0.7
Phenylalanine	C9H11NO2	n/a	n/a	**-0.40**	**3.98E-01**	165	156	**0.66**	63	Neutral-Catanionoeutral	0 IS 1+, 1-	Benzyl-CH2, NH3+, COO-	-0.6
Tryptophan	C11H12N2O2	n/a	n/a	**-0.30**	**5.01E-01**	204	138	**0.63**	79	Neutral-Catanionoeutral	0 IS 1+, 1-	Indole-CH2, NH3+, COO-	-0.5

Red = Not Permeable
Green = Permeable

TABLE 4B. Hydrophiles: Neutral-Catanionoeutral and Catanionoeutral through Inter-Epithelial Pore Complexes

	Formula	Log Pow	Pow	Log Dow	Dow	Weight (Daltons)	Volume (Ang3)	vdWD (nm)	Psa	Ionicity	Charge Distribution	Groups	HOWPC-to-vdWD Ratio (per nm [nm-1])
Asparagine	C4H7NO4	-4.50	3.16E-05	**-3.60**	**2.51E-04**	132	115	**0.60**	106	Neutral-Catanionoeutral	0 IS 1+, 1-	NH3 , NH3+, COO-	-6.0
Selenocysteineic Acid	C3H7NO2Se	n/a	n/a	**-3.25**	**5.62E-04**	168	108	**0.58**	106	Neutral-Catanionoeutral	0 IS 1+, 1-	HSe, NH3+, COO-	-5.6
Serine	C3H7NO3	n/a	n/a	**-3.10**	**7.94E-04**	105	94	**0.56**	84	Neutral-Catanionoeutral	0 IS 1+, 1-	OH, NH3+, COO-	-5.5
Glutamine	C5H10N2O3	n/a	n/a	**-3.30**	**5.01E-04**	146	132	**0.62**	63	Neutral-Catanionoeutral	0 IS 1+, 1-	NH2, NH3+, COO-	-5.3
Glycine	C2H5NO2	n/a	n/a	**-2.65**	**2.24E-03**	75	68	**0.50**	63	Catanionoeutral	IS 1+, 1-	NH3+, COO-	-5.3
Citruline	C6H13N3O3	n/a	n/a	**-3.20**	**6.31E-04**	175	161	**0.67**	118	Neutral-Catanionoeutral	0 IS 1+, 1-	NH2, NH3+, COO-	-4.8
Histidine @ pH 7.4	C6H9N3O2	n/a	n/a	**-2.90**	**1.26E-03**	155	135	**0.63**	92	Neutral-Catanionoeutral	0 IS 1+, 1-	NH2, NH3+, COO-	-4.6
Threonine	C4H9NO3	n/a	n/a	**-2.70**	**2.00E-03**	119	111	**0.59**	84	Neutral-Catanionoeutral	0 IS 1+, 1-	OH, NH3+, COO-	-4.6
Alanine	C3H7NO2	n/a	n/a	**-2.10**	**7.94E-03**	89	84	**0.54**	63	Neutral-Catanionoeutral	0 IS 1+, 1-	CH3, NH3+, COO-	-3.9
Taurine	C2H7NO3S	n/a	n/a	**-2.10**	**7.94E-03**	125	102	**0.57**	80	Catanionoeutral	IS 1+, 1-	NH3+, CH2-COO-	-3.7
Cysteine	C3H7NO2S	n/a	n/a	**-1.95**	**1.12E-02**	121	103	**0.57**	63	Neutral-Catanionoeutral	0 IS 1+, 1-	SH, NH3+, COO-	-3.4
Proline	C5H9NO2	n/a	n/a	**-1.75**	**1.78E-02**	115	108	**0.58**	49	Neutral-Catanionoeutral	0 IS 1+, 1-	NH2+ (Cyclopentane), COO-	-3.0
Methionine	C5H11NO2S	n/a	n/a	**-1.40**	**3.98E-02**	149	138	**0.63**	63	Neutral-Catanionoeutral	0 IS 1+, 1-	CH2-S-CH3, NH3+, COO-	-2.2
Valine	C5H11NO2	n/a	n/a	**-1.10**	**7.94E-02**	117	119	**0.60**	63	Neutral-Catanionoeutral	0 IS 1+, 1-	CH3 X2, NH3+, COO-	-1.8
ACPC	C6H11NO2	n/a	n/a	**-0.90**	**1.26E-01**	129	125	**0.61**	64	Neutral-Catanionoeutral	1 IS 1+, 1-	COO-, NH3+, Cyclopentane	-1.5
Levodopa (L-DOPA)	C9H11NO4	n/a	n/a	**-0.90**	**1.26E-01**	197	172	**0.68**	104	Neutral-Catanionoeutral	0 IS 1+, 1-	COO-, NH3+, Hydroxylated phenol	-1.3
Leucine	C6H13NO2	n/a	n/a	**-0.70**	**2.00E-01**	131	138	**0.63**	63	Neutral-Catanionoeutral	0 IS 1+, 1-	Isopropyl-CH3, NH3+, COO-	-1.1
Tyrosine	C9H11NO3	n/a	n/a	**-0.70**	**2.00E-01**	181	164	**0.67**	83	Neutral-Catanionoeutral	0 IS 1+, 1-	Benzyl-CH2-, OH X1, NH3+, COO-	-1.0
Isoleucine	C6H13NO2	n/a	n/a	**-0.60**	**2.51E-01**	131	136	**0.63**	63	Neutral-Catanionoeutral	0 IS 1+, 1-	Propyl-CH3, NH3+, COO-	-1.0
Pregabalin	C8H17NO2	n/a	n/a	**-0.55**	**2.82E-01**	159	170	**0.68**	63	Neutral-Catanionoeutral	0 IS 1+, 1-	COO-, NH3+, Isopropyl	-0.8
ACHC	C7H13NO2	n/a	n/a	**-0.50**	**3.16E-01**	143	142	**0.64**	63	Neutral-Catanionoeutral	0 IS 1+, 1-	COO-, NH3+, Cyclohexane	-0.8
Gabapentin	C9H17NO2	n/a	n/a	**-0.50**	**3.16E-01**	171	176	**0.69**	63	Neutral-Catanionoeutral	0 IS 1+, 1-	COO-, NH3+, Cyclohexane	-0.7
Phenylalanine	C9H11NO2	n/a	n/a	**-0.40**	**3.98E-01**	165	156	**0.66**	63	Neutral-Catanionoeutral	0 IS 1+, 1-	Benzyl-CH2, NH3+, COO-	-0.6
Tryptophan	C11H12N2O2	n/a	n/a	**-0.30**	**5.01E-01**	204	138	**0.63**	79	Neutral-Catanionoeutral	0 IS 1+, 1-	Indole-CH2, NH3+, COO-	-0.5

Red = Not Permeable
Green = Permeable
