# Supplementary material for: Permeation thresholds for hydrophilic small biomolecules across microvascular and epithelial barriers are predictable on basis of conserved biophysical properties
Source: In Silico Pharmacol. 2015 May 3;3:5. doi: 10.1186/s40203-015-0009-y (PMC4471070; doi:10.1186/s40203-015-0009-y)
Supplement: Additional file 5: Table S5. — Panel A. Hydrophiles: Mixed Polyneutral through Tight Junction Pore Complexes; Panel B. Mixed Hydrophiles: Mixed Polyneutral through Inter-Epithelial Pore Complexes. [file 40203_2015_9_MOESM5_ESM.pdf]

## TABLE 5A. Hydrophiles: Mixed Polyneutral through Tight Junction Pore Complexes

| | Formula | Log Pow | Pow | Log Dow | Dow | Weight (Daltons) | Volume (Ang3) | vdWD (nm) | Psa | Ionicity | Charge Distribution | Groups | HOWPC-to-vdWD Ratio (per nm [nm-1]) |
|---|---|---|---|---|---|---|---|---|---|---|---|---|---|
| Glucosamine @ Basic pH | C6H13NO5 | -3.09 | 8.128E-04 | n/a | n/a | 179 | 158 | 0.66 | 116 | Polyneutral | n/a | NH, OH X4 | -4.7 |
| Cytidine | C9H13N3O5 | -2.90 | 1.259E-03 | n/a | n/a | 243 | 201 | 0.72 | 129 | Polyneutral | n/a | OH X3, Ns | -4.0 |
| Guanosine | C10H13N5O5 | -2.71 | 1.950E-03 | n/a | n/a | 283 | 224 | 0.74 | 155 | Polyneutral | n/a | OH X3, Ns | -3.6 |
| Hydrogen cAscorbate Ester @ pH 2 | C6H8O6 | -1.91 | 1.23E-02 | n/a | n/a | 176 | 140 | 0.64 | 107 | Polyneutral | n/a | OH X4 | -3.0 |
| Adenosine | C10H13N5O4 | -2.09 | 8.128E-03 | n/a | n/a | 267 | 216 | 0.74 | 61 | Polyneutral | n/a | OH X3, Ns | -2.8 |
| Thymidine | C10H14N2O5 | -1.12 | 7.59E-02 | n/a | n/a | 242 | 206 | 0.72 | 99 | Polyneutral | n/a | OH X2, N X2, CH3 | -1.5 |

Red = Not Permeable

Green = Permeable

## TABLE 5B. Hydrophiles: Mixed Polyneutral through Inter-Epithelial Pore Complexes

| | Formula | Log Pow | Pow | Log Dow | Dow | Weight (Daltons) | Volume (Ang3) | vdWD (nm) | Psa | Ionicity | Charge Distribution | Groups | HOWPC-to-vdWD Ratio (per nm [nm-1]) |
|---|---|---|---|---|---|---|---|---|---|---|---|---|---|
| Glucosamine @ Basic pH | C6H13NO5 | -3.09 | 8.128E-04 | n/a | n/a | 179 | 158 | 0.66 | 116 | Polyneutral | n/a | NH, OH X4 | -4.7 |
| Cytidine | C9H13N3O5 | -2.90 | 1.259E-03 | n/a | n/a | 243 | 201 | 0.72 | 129 | Polyneutral | n/a | OH X3, Ns | -4.0 |
| Guanosine | C10H13N5O5 | -2.71 | 1.950E-03 | n/a | n/a | 283 | 224 | 0.74 | 155 | Polyneutral | n/a | OH X3, Ns | -3.6 |
| Hydrogen cAscorbate Ester @ pH 2 | C6H8O6 | -1.91 | 1.23E-02 | n/a | n/a | 176 | 140 | 0.64 | 107 | Polyneutral | n/a | OH X4 | -3.0 |
| Adenosine | C10H13N5O4 | -2.09 | 8.128E-03 | n/a | n/a | 267 | 216 | 0.74 | 61 | Polyneutral | n/a | OH X3, Ns | -2.8 |
| Thymidine | C10H14N2O5 | -1.12 | 7.59E-02 | n/a | n/a | 242 | 206 | 0.72 | 99 | Polyneutral | n/a | OH X2, N X2, CH3 | -1.5 |

Red = Not Permeable

Green = Permeable
