# Supplementary material for: Permeation thresholds for hydrophilic small biomolecules across microvascular and epithelial barriers are predictable on basis of conserved biophysical properties
Source: In Silico Pharmacol. 2015 May 3;3:5. doi: 10.1186/s40203-015-0009-y (PMC4471070; doi:10.1186/s40203-015-0009-y)
Supplement: Additional file 6: Table S6. — Panel A. Hydrophiles: Neutral through Tight Junction Pore Complexes; Panel B. Hydrophiles: Neutral through Inter-Epithelial Pore Complexes. [file 40203_2015_9_MOESM6_ESM.pdf]

TABLE 6A. Hydrophiles: Neutral through Tight Junction Pore Complexes

	Formula	Log Pow	Pow	Log Dow	Dow	Weight (Daltons)	Volume (Ang3)	vdWD (nm)	Psa	Ionicity	Charge Distribution	Groups	HOWPC-to-vdWD Ratio (per nm [nm-1])
Urea	CH4N2O	-1.36	4.365E-02	n/a	n/a	60	53	0.46	69	Neutral	n/a	NH2 X2	-3.0
Formamide	CH3NO	-1.11	7.762E-02	n/a	n/a	45	42	0.43	43	Neutral	n/a	H-C=O, N	-2.6
Cytosine	C4H5N3O	-1.40	3.981E-02	n/a	n/a	111	92	0.55	67	Neutral	n/a	N X2, NH2 X1	-2.5
Creatinine	C4H7N3O	-1.06	8.710E-02	n/a	n/a	113	98	0.56	56	Neutral	n/a	N X2, NH X1	-1.9
Guanine	C5H5N5O	-1.00	1.000E-01	n/a	n/a	151	115	0.60	96	Neutral	n/a	N X4, NH2 X1	-1.7
Adenine	C5H5N5	-0.53	2.951E-01	n/a	n/a	135	107	0.58	80	Neutral	n/a	N X4, NH2 X1	-0.9
Thymine	C5H6N2O2	-0.46	3.467E-01	n/a	n/a	126	106	0.58	58	Neutral	n/a	N X2	-0.8
51Cr-EDTA	C10H16CrN2O8	-0.43	3.715E-01	n/a	n/a	357	213	0.73	80	Neutral	n/a	Chelating COO- X4, Interior N+	-0.6
Methylthiourea	C2H6N2S	-0.30	5.012E-01	n/a	n/a	90	80	0.53	38	Neutral	n/a	S, NH2, CH3-NH	-0.6
Dioxygen	O2	-0.19	6.457E-01	n/a	n/a	32	22	0.34	34	Neutral	n/a	O2	-0.6
Carbon Dioxide	CO2	-0.20	6.310E-01	n/a	n/a	44	33	0.39	34	Neutral	n/a	CO2	-0.5
Ferrocyanide	C6FeN6	-0.26	5.495E-01	n/a	n/a	212	154	0.66	143	Neutral	n/a	Fe, CN X6	-0.4
Ethanol	C2H6O	-0.16	6.918E-01	n/a	n/a	46	54	0.46	20	Neutral	n/a	OH	-0.3

Red = Not Permeable
Green = Permeable

TABLE 6B. Hydrophiles: Neutral through Inter-Epithelial Pore Complexes

	Formula	Log Pow	Pow	Log Dow	Dow	Weight (Daltons)	Volume (Ang3)	vdWD (nm)	Psa	Ionicity	Charge Distribution	Groups	HOWPC-to-vdWD Ratio (per nm [nm-1])
Urea	CH4N2O	-1.36	4.365E-02	n/a	n/a	60	53	0.46	69	Neutral	n/a	NH2 X2	-3.0
Formamide	CH3NO	-1.11	7.762E-02	n/a	n/a	45	42	0.43	43	Neutral	n/a	H-C=O, N	-2.6
Cytosine	C4H5N3O	-1.40	3.981E-02	n/a	n/a	111	92	0.55	67	Neutral	n/a	N X2, NH2 X1	-2.5
Creatinine	C4H7N3O	-1.06	8.710E-02	n/a	n/a	113	98	0.56	56	Neutral	n/a	N X2, NH X1	-1.9
Guanine	C5H5N5O	-1.00	1.000E-01	n/a	n/a	151	115	0.60	96	Neutral	n/a	N X4, NH2 X1	-1.7
Adenine	C5H5N5	-0.53	2.951E-01	n/a	n/a	135	107	0.58	80	Neutral	n/a	N X4, NH2 X1	-0.9
Thymine	C5H6N2O2	-0.46	3.467E-01	n/a	n/a	126	106	0.58	58	Neutral	n/a	N X2	-0.8
51Cr-EDTA	C10H16CrN2O8	-0.43	3.715E-01	n/a	n/a	357	213	0.73	80	Neutral	n/a	Chelating COO- X4, Interior N+	-0.6
Methylthiourea	C2H6N2S	-0.30	5.012E-01	n/a	n/a	90	80	0.53	38	Neutral	n/a	S, NH2, CH3-NH	-0.6
Dioxygen	O2	-0.19	6.457E-01	n/a	n/a	32	22	0.34	34	Neutral	n/a	O2	-0.6
Carbon Dioxide	CO2	-0.20	6.310E-01	n/a	n/a	44	33	0.39	34	Neutral	n/a	CO2	-0.5
Ferrocyanide	C6FeN6	-0.26	5.495E-01	n/a	n/a	212	154	0.66	143	Neutral	n/a	Fe, CN X6	-0.4
Ethanol	C2H6O	-0.16	6.918E-01	n/a	n/a	46	54	0.46	20	Neutral	n/a	OH	-0.3

Red = Not Permeable
Green = Permeable
