# Supplementary material for: Permeation thresholds for hydrophilic small biomolecules across microvascular and epithelial barriers are predictable on basis of conserved biophysical properties
Source: In Silico Pharmacol. 2015 May 3;3:5. doi: 10.1186/s40203-015-0009-y (PMC4471070; doi:10.1186/s40203-015-0009-y)
Supplement: Additional file 7: Table S7. — Panel A. Hydrophiles: Cationic-Cationoneutral through Tight Junction Pore Complexes; Panel B. Hydrophiles: Cationic- Cationoneutral through Inter-Epithelial Pore Complexes.) [file 40203_2015_9_MOESM7_ESM.pdf]

TABLE 7A. Hydrophiles: Cationic-Cataniononeutral through Tight Junction Pore Complexes

	Formula	Log Pow	Pow	Log Dow	Dow	Weight (Daltons)	Volume (Ang3)	vdWD (nm)	Psa	Ionicity	Charge Distribution	Groups	HOWPC-to-vdWD Ratio (per nm [nm-1])
**Arginine**	C6H14N4O2	-3.16	6.92E-04	**-5.00**	**1.00E-05**	174	163	**0.67**	125	**Cationic-Catanioneutral**	**S 1+ IS 1+, 1-**	**NH2+, NH3+, COO-**	**-7.5**
**Lysine**	C6H14N2O2	-3.21	6.17E-04	**-4.75**	**1.78E-05**	146	148	**0.65**	89	**Cationic-Catanioneutral**	**S 1+ IS 1+, 1-**	**NH3+, NH3+, COO-**	**-7.3**
**Histidine @ pH <7.4**	C6H9N3O2	-2.90	1.26E-03	**-3.60**	**2.51E-04**	155	135	**0.63**	92	**Cationic-Catanioneutral**	**S 1+ IS 1+, 1-**	**NH+, NH3+, COO-**	**-5.7**

Red = Not Permeable
Green = Permeable

TABLE 7B. Hydrophiles: Cationic-Catanioneutral through Inter-Epithelial Pore Complexes

	Formula	Log Pow	Pow	Log Dow	Dow	Weight (Daltons)	Volume (Ang3)	vdWD (nm)	Psa	Ionicity	Charge Distribution	Groups	HOWPC-to-vdWD Ratio (per nm [nm-1])
**Arginine**	C6H14N4O2	-3.16	6.92E-04	**-5.00**	**1.00E-05**	174	163	**0.67**	125	**Cationic-Catanioneutral**	**S 1+ IS 1+, 1-**	**NH2+, NH3+, COO-**	**-7.5**
**Lysine**	C6H14N2O2	-3.21	6.17E-04	**-4.75**	**1.78E-05**	146	148	**0.65**	89	**Cationic-Catanioneutral**	**S 1+ IS 1+, 1-**	**NH3+, NH3+, COO-**	**-7.3**
**Histidine @ pH < 7.4**	C6H9N3O2	-2.90	1.26E-03	**-3.60**	**2.51E-04**	155	135	**0.63**	92	**Cationic-Catanioneutral**	**S 1+ IS 1+, 1-**	**NH+, NH3+, COO-**	**-5.7**

Red = Not Permeable
Green = Permeable
