# Supplementary material for: Permeation thresholds for hydrophilic small biomolecules across microvascular and epithelial barriers are predictable on basis of conserved biophysical properties
Source: In Silico Pharmacol. 2015 May 3;3:5. doi: 10.1186/s40203-015-0009-y (PMC4471070; doi:10.1186/s40203-015-0009-y)
Supplement: Additional file 8: Table S8. — Panel A. Hydrophiles: Cationic through Tight Junction Pore Complexes; Panel B. Hydrophiles: Cationic through Inter-Epithelial Pore Complexes. [file 40203_2015_9_MOESM8_ESM.pdf]

TABLE 8A. Hydrophiles: Cationic through Tight Junction Pore Complexes

	Formula	Log Pow	Pow	Log Dow	Dow	Weight (Daltons)	Volume (Ang3)	vdWD (nm)	Psa	Ionicity	Charge Distribution	Groups	HOWPC-to-vdWD Ratio (per nm [nm-1])
**Glucosamine @ Acidic pH**	C6H13NO5	n/a	n/a	**-5.75**	**1.778E-06**	179	158	**0.66**	116	Cationic	1+	NH2+, OH X4	-8.7
**Methylammonium**	CH6N	n/a	n/a	**-3.20**	**6.310E-04**	32	43	**0.43**	27	Cationic	1+	CH3-NH3+	-7.5
**Choline**	C5H14NO	n/a	n/a	**-4.20**	**6.31E-05**	104	121	**0.61**	20	Cationic	1+	Quat N+, OH	-6.9
**Dimethylammonium**	C2H8N	n/a	n/a	**-3.00**	**1.000E-03**	46	60	**0.48**	17	Cationic	1+	(CH3)2-NH2+	-6.3
**Tetramethylammonium (TMA)**	C4H12N	n/a	n/a	**-3.50**	**3.162E-04**	74	96	**0.56**	0	Cationic	1+	Quat N+ [(CH3)4-N+]	-6.2
**Acetylcholine Ester**	C7H16NO2	n/a	n/a	**-3.72**	**1.91E-04**	146	158	**0.66**	26	Cationic	1+	Quat N+, OCH3	-5.6
**Tropine**	C8H15NO	n/a	n/a	**-3.00**	**1.000E-03**	141	146	**0.65**	23	Cationic	1+	(CH3)3N+, OH	-4.6
**Muscurine Ester (Muscurine)**	C9H20NO2	n/a	n/a	**-2.50**	**3.162E-03**	174	187	**0.70**	29	Cationic	1+	Quat N+, OCH3	-3.6
**Tetraethylammonium (TEA)**	C8H20N	n/a	n/a	**-2.06**	**8.710E-03**	130	164	**0.67**	0	Cationic	1+	(CH3CH2)4-N+	-3.1
**Edrophonium**	C10H16NO	n/a	n/a	**-1.20**	**6.310E-02**	166	175	**0.69**	20	Cationic	1+	Quat N+, OH	-1.8
**4-Aminopyridine**	C5H6N2	n/a	n/a	**-0.57**	**2.692E-01**	94	89	**0.55**	39	Cationic	1+	(CH2)N+(CH), NH2	-1.0

Red = Not Permeable
Green = Permeable

TABLE 8B. Hydrophiles: Cationic through Inter-Epithelial Pore Complexes

	Formula	Log Pow	Pow	Log Dow	Dow	Weight (Daltons)	Volume (Ang3)	vdWD (nm)	Psa	Ionicity	Charge Distribution	Groups	HOWPC-to-vdWD Ratio (per nm [nm-1])
**Glucosamine @ Acidic pH**	C6H13NO5	n/a	n/a	**-5.75**	**1.778E-06**	179	158	**0.66**	116	Cationic	1+	NH2+, OH X4	-8.7
**Methylammonium**	CH6N	n/a	n/a	**-3.20**	**6.310E-04**	32	43	**0.43**	27	Cationic	1+	CH3-NH3+	-7.5
**Choline**	C5H14NO	n/a	n/a	**-4.20**	**6.31E-05**	104	121	**0.61**	20	Cationic	1+	Quat N+, OH	-6.9
**Dimethylammonium**	C2H8N	n/a	n/a	**-3.00**	**1.000E-03**	46	60	**0.48**	17	Cationic	1+	(CH3)2-NH2+	-6.3
**Tetramethylammonium (TMA)**	C4H12N	n/a	n/a	**-3.50**	**3.162E-04**	74	96	**0.56**	0	Cationic	1+	Quat N+ [(CH3)4-N+]	-6.2
**Acetylcholine Ester**	C7H16NO2	n/a	n/a	**-3.72**	**1.91E-04**	146	158	**0.66**	26	Cationic	1+	Quat N+, OCH3	-5.6
**Tropine**	C8H15NO	n/a	n/a	**-3.00**	**1.000E-03**	141	146	**0.65**	23	Cationic	1+	(CH3)3N+, OH	-4.6
**Muscurine Ester (Muscurine)**	C9H20NO2	n/a	n/a	**-2.50**	**3.162E-03**	174	187	**0.70**	29	Cationic	1+	Quat N+, OCH3	-3.6
**Tetraethylammonium (TEA)**	C8H20N	n/a	n/a	**-2.06**	**8.710E-03**	130	164	**0.67**	0	Cationic	1+	(CH3CH2)4-N+	-3.1
**Edrophonium**	C10H16NO	n/a	n/a	**-1.20**	**6.310E-02**	166	175	**0.69**	20	Cationic	1+	Quat N+, OH	-1.8
**4-Aminopyridine**	C5H6N2	n/a	n/a	**-0.57**	**2.692E-01**	94	89	**0.55**	39	Cationic	1+	(CH2)N+(CH), NH2	-1.0

Red = Not Permeable
Green = Permeable
