# Supplementary material for: Permeation thresholds for hydrophilic small biomolecules across microvascular and epithelial barriers are predictable on basis of conserved biophysical properties
Source: In Silico Pharmacol. 2015 May 3;3:5. doi: 10.1186/s40203-015-0009-y (PMC4471070; doi:10.1186/s40203-015-0009-y)
Supplement: Additional file 9: Table S9. — Panel A. Hydrophiles: Cationic-Anionic through Tight Junction Pore Complexes; Panel B. Hydrophiles: Cationic-Anionic through Inter-Epithelial Pore Complexes. [file 40203_2015_9_MOESM9_ESM.pdf]

TABLE9A. Hydrophiles: Cationic-Anionic through Tight Junction Pore Complexes

	Formula	Log Pow	Pow	Log Dow	Dow	Weight (Daltons)	Volume (Ang3)	vdWD (nm)	Psa	Ionicity	Charge Distribution	Groups	HOWPC-to-vdWD Ratio (per nm [nm-1])
Creatine Phosphate	C4H10N3O5P	-2.25	5.62E-03	**-8.00**	**1.000E-08**	211	163	**0.67**	134	**Cationic-Polyanionic**	**PS 1+, 1-(1-) 1-**	**NH2+, PO3 2-,COO-**	**-12.0**
Phosphocholine	C5H14NO4P	-2.30	5.01E-03	**-4.80**	**1.585E-05**	183	164	**0.67**	70	**Cationic-PolyAnionic**	**PS 1+, 1-(1-)**	***Quat N+, PO4 2-**	**-7.2**
Phosphoethanolamine	C2H8NO4P	-3.40	3.98E-04	**-4.10**	**7.943E-05**	141	110	**0.59**	104	**Cationic-PolyAnionic**	**PS 1+, 1-(1-)**	**NH3+, PO3 2-**	**-7.0**
Tetrodotoxin	C11H17N3O8	-4.90	1.259E-05	**-4.90**	**1.259E-05**	319	252	**0.77**	187	**Cationic-Anionic**	**PS 1+, 1-**	**NH2+, O-, OHs**	**-6.3**
Gamma-aminobutyric acid (GABA)	C4H9NO2	-2.10	7.94E-03	**-2.10**	**7.94E-03**	103	102	**0.57**	63	**NR Cationic-Anionic**	**NR PS 1+, 1-**	**NH3[+]-CH2-CH2-CH2-COO-**	**-3.7**

Red = Not Permeable

Green = Permeable

TABLE9B. Hydrophiles: Cationic-Anionic through Adherens Junction Pore Complexes

	Formula	Log Pow	Pow	Log Dow	Dow	Weight (Daltons)	Volume (Ang3)	vdWD (nm)	Psa	Ionicity	Charge Distribution	Groups	HOWPC-to-vdWD Ratio (per nm [nm-1])
Creatine Phosphate	C4H10N3O5P	-2.25	5.62E-03	**-8.00**	**1.000E-08**	211	163	**0.67**	134	**Cationic-Polyanionic**	**PS 1+, 1-(1-) 1-**	**NH2+, PO3 2-,COO-**	**-12.0**
Phosphocholine	C5H14NO4P	-2.30	5.01E-03	**-4.80**	**1.585E-05**	183	164	**0.67**	70	**Cationic-PolyAnionic**	**PS 1+, 1-(1-)**	***Quat N+, PO4 2-**	**-7.2**
Phosphoethanolamine	C2H8NO4P	-3.40	3.98E-04	**-4.10**	**7.943E-05**	141	110	**0.59**	104	**Cationic-PolyAnionic**	**PS 1+, 1-(1-)**	**NH3+, PO3 2-**	**-7.0**
Tetrodotoxin	C11H17N3O8	-4.90	1.259E-05	**-4.90**	**1.259E-05**	319	252	**0.77**	187	**Cationic-Anionic**	**PS 1+, 1-**	**NH2+, O-, OHs**	**-6.3**
Gamma-aminobutyric acid (GABA)	C4H9NO2	-2.10	7.94E-03	**-2.10**	**7.94E-03**	103	102	**0.57**	63	**NR Cationic-Anionic**	**NR PS 1+, 1-**	**NH3[+]-CH2-CH2-CH2-COO-**	**-3.7**

Red = Not Permeable

Green = Permeable
