# Supplementary material for: Permeation thresholds for hydrophilic small biomolecules across microvascular and epithelial barriers are predictable on basis of conserved biophysical properties
Source: In Silico Pharmacol. 2015 May 3;3:5. doi: 10.1186/s40203-015-0009-y (PMC4471070; doi:10.1186/s40203-015-0009-y)
Supplement: Additional file 10: Table S10. — Panel A. Endogenous and Non-endogenous Non-Metals and Metals through Zona Occludens Tight Junction Pore Complexes; Panel B. Endogenous and Non-endogenous Non-Metals and Metals through Inter-Epithelial Pore Complexes. [file 40203_2015_9_MOESM10_ESM.pdf]

TABLE 10A. Endogenous and Non-endogenous Non-Metals and Metals through Zona Occludens Tight Junction Pore Complexes

Cation	Mass (daltons)	Radius (nm)	Diameter (nm)	Cationization (+)	CI-to-AD Ratio (nm-1)
K+	39	0.227	0.454	1	+2.20
CH3-Hg+&	216	0.210	0.420	1	+2.38
Na+	23	0.186	0.372	1	+2.69
Ba2+	137	0.222	0.444	2	+4.50
Sr2+	88	0.215	0.430	2	+4.65
Ca2+	40	0.197	0.394	2	+5.08
Pb2+	207	0.175	0.350	2	+5.71
Mg2+	24	0.160	0.320	2	+6.25
Cd2+	112	0.151	0.302	2	+6.62
Hg2+	201	0.151	0.302	2	+6.62
Zn2+	65	0.134	0.268	2	+7.46
Cu2+	64	0.128	0.256	2	+7.81
Mn2+	55	0.127	0.254	2	+7.87
Fe2+	56	0.126	0.252	2	+7.94
Co2+	59	0.125	0.250	2	+8.00
Ni2+	59	0.124	0.248	2	+8.06
Be2+	9	0.112	0.224	2	+8.93
Cr3+	52	0.128	0.256	3	+11.72
Fe3+	56	0.126	0.252	3	+11.90
As3+	75	0.119	0.238	3	+12.61

&CH3-Hg+, a compact heavy metal organic, is appropriately grouped with the non-organic elemental forms, as Hg is dense metal, with the CH3 close to its center of gravity as is its cationic charge, where its overall diameter is disproportionately influenced by its atomic weight than that of CH3's

Green = permeable
Red = Not permeable

TABLE 10B. Endogenous and Non-endogenous Non-Metals and Metals through Inter-Epithelial Pore Complexes

Cation	Mass (daltons)	Radius (nm)	Diameter (nm)	Cationization (+)	CI-to-AD Ratio (nm-1)
K+	39	0.227	0.454	1	+2.20
CH3-Hg+&	216	0.210	0.420	1	+2.38
Na+	23	0.186	0.372	1	+2.69
Ba2+	137	0.222	0.444	2	+4.50
Sr2+	88	0.215	0.430	2	+4.65
Ca2+#	40	0.197	0.394	2	+5.08
Pb2+	207	0.175	0.350	2	+5.71
Mg2+	24	0.160	0.320	2	+6.25
Cd2+	112	0.151	0.302	2	+6.62
Hg2+	201	0.151	0.302	2	+6.62
Zn2+	65	0.134	0.268	2	+7.46
Cu2+	64	0.128	0.256	2	+7.81
Mn2+	55	0.127	0.254	2	+7.87
Fe2+	56	0.126	0.252	2	+7.94
Co2+	59	0.125	0.250	2	+8.00
Ni2+	59	0.124	0.248	2	+8.06
Be2+	9	0.112	0.224	2	+8.93
Cr3+	52	0.128	0.256	3	+11.72
Fe3+	56	0.126	0.252	3	+11.90
As3+	75	0.119	0.238	3	+12.61

&CH3-Hg+, a compact heavy metal organic, is appropriately grouped with the non-organic elemental forms,
#Ca2+ is partially permeable across adherens loose junctions as Hg is dense metal, with the CH3 close to its center of gravity as is its cationic charge, where its overall diameter is disproportionately influenced by its atomic weight than that of CH3's

Green = permeable
Red = Not permeable
