# Supplementary material for: Permeation thresholds for hydrophilic small biomolecules across microvascular and epithelial barriers are predictable on basis of conserved biophysical properties
Source: In Silico Pharmacol. 2015 May 3;3:5. doi: 10.1186/s40203-015-0009-y (PMC4471070; doi:10.1186/s40203-015-0009-y)
Supplement: Additional file 11: Table S11. — Panel A. Biological Halogens through Zona Occludens Tight Junction Pore Complexes; Panel B. Biological Halogens through Inter-Epithelial Pore Complexes. [file 40203_2015_9_MOESM11_ESM.pdf]

TABLE 11A. Biological Halogens through Zona Occludens Tight Junction Pore Complexes

Anion	Mass (daltons)	Radius (nm)	Diameter (nm)	Anionization (-)	AI-to-AD Ratio (nm-1)
I-	127	0.140	0.280	1	-3.57
Br-	80	0.120	0.240	1	-4.17
Cl-	36	0.102	0.204	1	-4.90
F-	19	0.064	0.128	1	-7.81

Border = Permeable

TABLE 11B. Biological Halogens through Inter-Epithelial Pore Complexes

Anion	Mass (daltons)	Radius (nm)	Diameter (nm)	Anionization (-)	AI-to-AD Ratio (nm-1)
I-	127	0.140	0.280	1	-3.57
Br-	80	0.120	0.240	1	-4.17
Cl-	36	0.102	0.204	1	-4.90
F-	19	0.064	0.128	1	-7.81

Border = Permeable
