# Supplementary material for: Permeation thresholds for hydrophilic small biomolecules across microvascular and epithelial barriers are predictable on basis of conserved biophysical properties
Source: In Silico Pharmacol. 2015 May 3;3:5. doi: 10.1186/s40203-015-0009-y (PMC4471070; doi:10.1186/s40203-015-0009-y)
Supplement: Additional file 12: Table S12. — Permeation Thresholds for Hydrophile Small Biomolecules across Zona Occludens Tight Junction Pore Complexes. [file 40203_2015_9_MOESM12_ESM.pdf]

TABLE 12. Permeation Thresholds for Hydrophiles across Zona Occludens Tight Junction Pore Complexes

	HOWPC-to-vdWD Ratio for Permeable Hydrophile (per nm [nm-1])	HOWPC-to-vdWD Ratio at Non-permeability (per nm [nm-1])	vdWD for Permeable Hydrophile @ MAXimum HOWPC-to-vdWD (nm)	vdWD for Permeable Hydrophile @ MINimum HOWPC-to-vdWD (nm)
Anionic	-5.7	-6.7	0.50	< 0.63
Anionic-Cataniononeutral	*CE*	*CE*	*CE*	n/a
Pure Polyneutral	-4.2	-4.5	0.59	n/a
Neutral-Catanionoenutral & Catanionoenutral	-3.9	-4.6	0.63	>= 0.69
Mixed Polyneutral	-3.0	n/a	n/a	>= 0.64 < 0.72
Neutral	-3.0	n/a	n/a	>= 0.66 < 0.73
Cationic-Catanionoenutral	*CE*	*CE*	*CE*	n/a
Cationic	-1.0	-1.8	0.55	DNE
Cationic-Anionic	-3.7	-6.3	0.57	DNE

CE = Charge Excluded

n/a = not applicable (see Results Section)

DNE = does not exist (see Results Section)
