# Supplementary material for: Permeation thresholds for hydrophilic small biomolecules across microvascular and epithelial barriers are predictable on basis of conserved biophysical properties
Source: In Silico Pharmacol. 2015 May 3;3:5. doi: 10.1186/s40203-015-0009-y (PMC4471070; doi:10.1186/s40203-015-0009-y)
Supplement: Additional file 13: Table S13. — Permeation Thresholds for Hydrophile Small Biomolecules across Inter-Epithelial Pore Complexes. [file 40203_2015_9_MOESM13_ESM.pdf]

**TABLE 13. Permeation Thesholds for Hydrophiles across Inter-Epithelial Junction Pore Complexes**

| | HOWPC-to-vdWD Ratio for Permeable Hydrophile (per nm [nm-1]) | HOWPC-to-vdWD Ratio at Non-permeability (per nm [nm-1]) | vdWD for Permeable Hydrophile @ MAXimum HOWPC-to-vdWD (nm) | vdWD for Permeable Hydrophile @ MINimum HOWPC-to-vdWD (nm) |
|---|---|---|---|---|
| Anionic | -8.4 | -8.5 | 0.48 | >0.78 |
| Anionic-Cataniononeutral | -7.0 | -8.7 | 0.71 | DNE |
| Pure Polyneutral | -5.6 | -6.7 | 0.81 | n/a |
| Neutral-Cataniononeutral & Cataniononeutral | -6.0 | n/a | n/a | >= 0.69 |
| Mixed Polyneutral | -4.7 | n/a | n/a | >= 0.74 |
| Neutral | -3.0 | n/a | n/a | >= 0.73 |
| Cationic-Cataniononeutral | *CE* | *CE* | *CE* | n/a |
| Cationic | -7.5 | -8.7 | 0.43 | >= 0.70 |
| Cationic-Anionic | -7.2 | -12.0 | 0.67 | >= 0.77 |

*CE = Charge Excluded*
n/a = not applicable (see Results Section)
DNE = does not exist (see Results Section)
