# Supplementary material for: Permeation thresholds for hydrophilic small biomolecules across microvascular and epithelial barriers are predictable on basis of conserved biophysical properties
Source: In Silico Pharmacol. 2015 May 3;3:5. doi: 10.1186/s40203-015-0009-y (PMC4471070; doi:10.1186/s40203-015-0009-y)
Supplement: Additional file 14: Table S14. — Permeation Thresholds of Pore Sizes for Hydrophilic Biomolecules across Blood Capillary Wall Endothelia in Nanometers. [file 40203_2015_9_MOESM14_ESM.pdf]

TABLE 14. Permeation Thresholds of Pore Sizes for Hydrophilic Biomolecules across Blood Capillary Wall Endothelia in Nanometers

	Diaphragm Fenestrated Abnormal (i.e. Solid Tumor)	Diaphragm Fenestrated Normal (i.e. Endocrine, Choriod Plexus, Renal Tubular)	Open Fenestrated Normal (Renal Glomerular&)	Open Fenestrated Normal (Myeloid Bone Marrow)	Macula Occludens Junctions (i.e. Muscle Capillaries)	Zona Occudens Tight Junctions (i.e. Hepatic Capillaries) [This Study's Findings]	Inter-Epithelial Junctions (i.e. Choroid Plexus Epithelium) [This Study's Findings]
**Anionic**	**12**	**--**	**2**	**--**	--	**0.50; < 0.63**	**0.48; > 0.78**
**Neutral/Polyneutral**	**12**	**6**	**2.3**	**7**	**3.9**	**0.59; 0.66 - 0.73**	**0.81; >= 0.74**
**Cationic**	**>=14**	**--**	**3-4.5**	**--**	**4.5**	**0.55; n/a**	**0.66 ; >= 0.70**

n/a = not applicable

& = Podocyte fenestration pore size; whereas, for glomerular cappilaries, anionic-to-cationic macromolecular tracers localize from at the level of endothelial glycocalyx-to-level of outer basement membrane layer

Note1: 12 nm theshold based on Gd-DTPA-PAMAM dendrimer of anionically neutralized exterior with hydrodynamic diameters 11 nm and 13 nm; >= 14 threshold based on cationized ferritin with diameter 12.8 nm; 6 nm threshold based on myoglobin with diameter 5.5

Note2: Macromolecules with cationized exteriors exert cationotoxicity to the endothelium and permeate at larger sizes than counterpart macromolecules with anionic or neutral exteriors, while anionic and cationic small hydrophiles with un-opposed anionic or cationic charge are relatively charge-excluded from permeation across junction pore complexes (see Results section)
